# Dynamic allocation of orthogonal ribosomes facilitates uncoupling of co-expressed genes

**DOI:** 10.1038/s41467-018-02898-6

**Published:** 2018-02-15

**Authors:** Alexander P. S. Darlington, Juhyun Kim, José I. Jiménez, Declan G. Bates

**Affiliations:** 1 Warwick Integrative Synthetic Biology Centre, School of Engineering, University of Warwick, Coventry, CV4 7AL UK; 2 Faculty of Health and Medical Sciences, University of Surrey, Guildford, GU2 7XH UK

## Abstract

Introduction of synthetic circuits into microbes creates competition between circuit and host genes for shared cellular resources, such as ribosomes. This can lead to the emergence of unwanted coupling between the expression of different circuit genes, complicating the design process and potentially leading to circuit failure. By expressing a synthetic 16S rRNA with altered specificity, we can partition the ribosome pool into host-specific and circuit-specific activities. We show mathematically and experimentally that the effects of resource competition can be alleviated by targeting genes to different ribosomal pools. This division of labour can be used to increase flux through a metabolic pathway. We develop a model of cell physiology which is able to capture these observations and use it to design a dynamic resource allocation controller. When implemented, this controller acts to decouple genes by increasing orthogonal ribosome production as the demand for translational resources by a synthetic circuit increases.

## Abstract (web summary)

Competition between synthetic genetic circuits and host genes for shared resources can complicate circuit design and lead to failure. Here the authors demonstrate, mathematically and experimentally, the use of orthogonal ribosomes to decouple competing genes.

## Introduction

A key goal of synthetic biology is the construction of novel information processing genetic circuits in microbes which can be used to guide cellular function and control metabolic processes^1^. However, many initial designs fail upon implementation in vivo due to unforeseen interactions between the host and synthetic circuit^2^. These often arise due to competition for shared cellular resources, such as RNA polymerases and ribosomes^3, 4^. Previous experimental studies have shown that translational capacity, in the form of free ribosomes, limits microbial gene expression^3, 5–8^ and so is a key cause of these hidden interactions^4^. Previous work has shown that the non-regulatory coupling that emerges due to these hidden interactions can be reduced by careful design and selection of ribosome binding sites and plasmid copy numbers^3, 4, 6^. Additionally, incorporating negative feedback loops into the circuit can insulate genes^9–11^. These approaches, however, require significant re-design of the original synthetic circuit, and by incorporating additional regulatory interactions may make certain circuit behaviours unobtainable. In this work, we propose an alternative approach, based on the partitioning of the cell’s translational capacity, and show that it allows the decoupling of circuit genes without the need for extensive re-design.

Previously, both transcription and protein degradation activities have been partitioned into circuit-specific and host-specific activities through the use of ‘orthogonal’ components. For example, the expression of RNA polymerases (RNAP) from bacteriophage T7 in *Escherichia coli* creates a circuit-specific transcription system^12^. Co-option of proteases from other bacteria has been used to create a circuit-specific degradation pathway^13^. Due to the universality and complexity of the cell’s translational machinery, there does not exist a sufficiently distinct ribosome which can be co-opted into *E. coli* to create a truly orthogonal ribosome pool. However, translational capacity can be divided into host- and circuit-specific functions by the use of synthetic ribosomal components to create a quasi-orthogonal ribosome (‘o-ribosome’) system^14–16^. The binding interactions between an mRNA and the 16S ribosomal RNA (rRNA) of the small ribosomal subunit are known to be a key regulator of translation initiation^17^, and thus o-ribosomes can be created by expressing a synthetic 16S rRNA. Evolving or designing the 5′ sequences at and around the ribosome binding site (RBS) of circuit mRNAs to interact with this synthetic sequence allows the creation of an orthogonal translation system^15, 16^. For simplicity, we refer to this synthetic 5′ sequence as an orthogonal RBS (‘o-RBS’). These specialised o-ribosomes have previously been successfully used to probe various aspects of ribosome function^18^ or for biocontainment^19^ but their use in the creation of synthetic gene circuits and managing the distribution of translational activity has not yet been fully explored.

In this paper, we demonstrate the use of orthogonal ribosomes for reducing coupling between the expression of different circuit genes. We use the division of translational activity provided by orthogonal ribosomes to design simple resource allocators, where circuit genes are targeted to either the host or orthogonal ribosome pools. We show that this can be used to relieve the effects of resource-mediated gene coupling and that by allocating circuit genes in this way flux though a metabolic pathway can be improved. We design a feedback controller that acts to dynamically increase o-ribosome production as demand for translational resources by the circuit genes increases. When implemented experimentally, this controller acts to reduce resource-mediated gene coupling by 50%. We develop a mathematical model of microbial growth, and show throughout that this can be used to assess how circuit genes should be allocated between different translational pools.

## Results

### Development of an o-ribosome model

We initially developed a mathematical model to assess the feasibility of implementing and using an orthogonal translation system. Taking a host-aware modelling approach we based our model on that of microbial physiology developed by Weiße et al.^20^. This model captures the three fundamental trade-offs in bacterial gene expression: (i) internal anabolic capacity (‘energy’) is limited by substrate import and enzyme activity, (ii) finite translational capacity and (iii) finite proteome size. This model consists of a simple metabolism, transcriptome and proteome representing four main class of protein function: (i) metabolite transport (*T*), (ii) enzymes (*E*), (iii) ribosomes (*R*) and (iv) host proteins (*H*). We refer to these host genes as *X* ∈ {*T*, *E*, *H*, *R*}. Additional proteins representing circuit genes, the set *Y*, are included as described in Supplementary Note 1. See Supplementary Fig. 1 for a simplified schematic. We fit our model to the growth rate and ribosomal mass fraction data as produced by Scott et al.^5^ (Supplementary Fig. 2).

We expanded on the original ribosome biosynthesis reaction to take account of both protein and rRNA components. We considered the production of a single large ribosomal protein to represent the small and large ribosomal subunits and other accessory protein complexes. We term this species the ‘empty ribosome’ (*p*_*R*_). This is encoded by an mRNA (*m*_*R*_), which is born spontaneously at a rate proportional to the cell’s internal ‘energy’ (Eq. (1)).

1 $$\begin{array}{*{20}{c}} {\emptyset \mathop{\longrightarrow}\limits^{{T_X(e)}}m_R} & {\emptyset \mathop{\longrightarrow}\limits^{{T_X(e)}}r} \end{array}.$$

The mRNA is translated by host ribosomes (*T*_*L*_ function), in the same manner as host proteins to produce the protein intermediate *p*_*R*_ (Eq. (2)).

2 $$m_R + R\begin{array}{*{20}{c}} {b_R} \\ \rightleftharpoons \\ {u_R} \end{array}c_R\mathop{\longrightarrow}\limits^{{T_L\left( {c_R,e} \right)}}m_R + R + p_R.$$

The host rRNA component (*r*) is also born spontaneously at a rate proportional to the cell’s internal ‘energy’ (*T*_*X*_ function) (Eq. (1)). This then binds empty ribosomes to form free host ribosomes (*R*); to account for ribosome complex disassembly, we assume this reaction is reversible (Eq. (3)).

3 $$p_R + r\begin{array}{*{20}{c}} {b_r} \\ \rightleftharpoons \\ {u_r} \end{array}R.$$

All components are subject to degradation (*δ* terms) and dilution (*λ*). Additionally, the host ribosome translates host proteins and circuit genes (the set *Y*) (Σ term in Eq. (7)).

To model the production of orthogonal ribosomes we introduce two new species, *ρ* representing the o-16S rRNA and *P* representing the o-ribosomes and the following reactions:

4 $$\begin{array}{*{20}{c}} {\emptyset \mathop{\longrightarrow}\limits^{{T_X(e)}}\rho } & {p_R + \rho \begin{array}{*{20}{c}} {b_\rho } \\ \rightleftharpoons \\ {u_\rho } \end{array}P} \end{array}.$$

We assume that the o-16S rRNA follows the same dynamics as the host rRNA but that this plasmid-carried gene will respond to energy changes in a similar manner to host genes—resulting in a description of the same form but a different parameterisation (Supplementary Tables 1 and 2).

Applying the law of mass action to the reactions involving the host and orthogonal 16S rRNA yields the following rate equations:

5 $$\frac{{{\rm d}r}}{{{\rm d}t}} = T_X(e) - b_r \cdot p_R \cdot r + u_r \cdot R - (\delta _r + \lambda ) \cdot r,$$

6 $$\frac{{{\rm d}\rho }}{{{\rm d}t}} = T_X(e) - b_\rho \cdot p_R \cdot \rho + u_\rho \cdot P - \left( {\delta _p + \lambda } \right) \cdot \rho.$$

The functional host, *R*, and orthogonal, *P*, ribosomes have the following dynamics:

7 $$\begin{array}{*{20}{l}} {\frac{{{\rm d}R}}{{{\rm d}t}}} \hfill & = \hfill & {b_r \cdot p_R \cdot r - u_r \cdot R} \hfill \\ {} \hfill & {} \hfill & { - \mathop {\sum}\limits_{X \in \{ T,E,H,R,Y\} } \left( {T_L\left( {c_X,e} \right) - b_X \cdot R \cdot m_X + u_X \cdot c_X} \right)} \hfill \\ {} \hfill & {} \hfill & { - \left( {\delta _R + \lambda } \right) \cdot R} \hfill \end{array},$$

8 $$\begin{array}{*{20}{l}} {\frac{{{\rm d}P}}{{{\rm d}t}}} \hfill & = \hfill & {b_\rho \cdot p_R \cdot u_\rho \cdot P} \hfill \\ {} \hfill & {} \hfill & { - \mathop {\sum}\limits_Y \left( {T_L\left( {c_Y,e} \right) - b_Y \cdot P \cdot m_Y + u_Y \cdot c_Y} \right)} \hfill \\ {} \hfill & {} \hfill & { - (\delta _R + \lambda ) \cdot P} \hfill \end{array}.$$

Note that the orthogonal ribosomes only translate circuit genes *Y* and not host proteins. The host pool can translate both host genes and circuit genes. The set *Y* can be distributed between both pools as described in Eq. (1).

The dynamics of the protein-based component *p*_*R*_ is given by:

9 $$\frac{{{\rm d}p_R}}{{{\rm d}t}} = T_L\left( {c_R,e} \right) - \left( {\delta _{p_R} + \lambda } \right) \cdot p_R - b_r \cdot p_R \cdot r + u_r \cdot R - b_\rho \cdot p_R \cdot \rho + u_\rho \cdot P.$$

By modelling ribosome biosynthesis in this way it incorporates the two important feedback loops which determine ribosome number: (i) host ribosomes are autocatalytic and (ii) ribosomal mRNA and rRNA transcription rates fall as ‘energy’ is consumed by protein production. It also incorporates the ‘quasi’-orthogonal nature of the o-ribosome pool by linking the two ribosome pools through competition for the protein-based components *p*_*R*_.

See Supplementary Note 1 for the full model derivation and description of additional reactions such as enzyme and host protein production. Our model predicts that cells can tolerate the use of orthogonal ribosomes for significant levels of gene expression (Supplementary Fig. 3a, b) and that partitioning of the ribosome pool can be used to divert translational machinery to a synthetic gene (Supplementary Fig. 3c).

### Construction of an orthogonal gene expression system in vivo

**Fig. 1:**
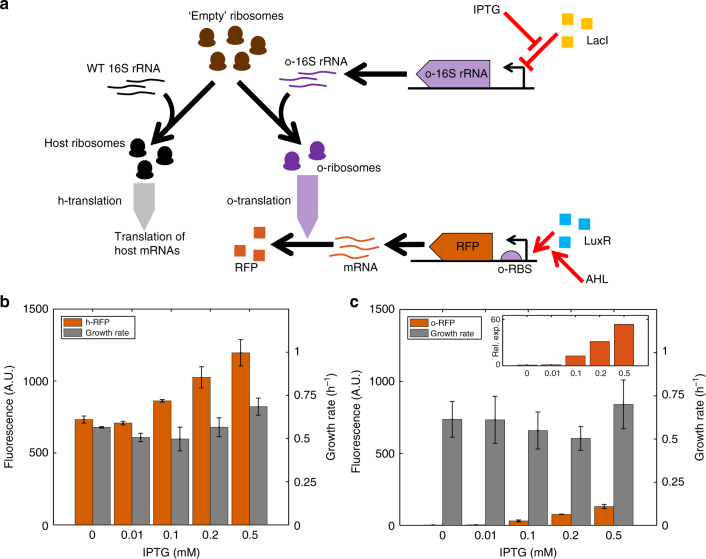
Developing the o-ribosome pool as an expression system. RFP expression using either the host or o-ribosome pool. o-ribosomes are produced by inducing o-16S rRNA with increasing concentrations of IPTG. (o/h)-RFP is induced with 20 nM AHL throughout. Bars represent means ± 1 SD. *N* = 3. **a** Schematic of the orthogonal translation system. **b** Expression of h-RFP by the host ribosome pool. **c** Expression of o-RFP by the o-ribosome pool. Inset, relative o-RFP expression. RFP levels normalised by background. *x*-axis, IPTG concentration (mM)

We utilised a previously described o-16S rRNA system, which contains an o-16S rRNA under the control of *P*_lac_, thus allowing its levels to be controlled by isopropyl β-D-1-thiogalactopyranoside (IPTG)^21^. Our circuit is carried on a second plasmid and consists of RFP under the control of the *P*_lux_ promoter. Translation by either the host (h-RFP) or orthogonal (o-RFP) ribosome pools is controlled by selection of the RBS (Fig. 1a). RFP mRNA production is induced with *N*-acyl homoserine lactone (AHL) via LuxR which is constitutively expressed from the circuit plasmid and utilises host ribosomes for its expression.

To assess the impact of o-ribosome production on the host growth rate and gene expression, gene induction was maintained using a constant concentration of AHL in the presence of increasing IPTG concentration. The production of o-ribosomes alone has no effect on growth rate demonstrating that their presence is non-toxic (Supplementary Fig. 4). We observe a 50% increase in h-RFP fluorescence (*p* < 0.05, *t*-test, 0 mM vs. 0.5 mM IPTG) (Fig. 1b). This is likely due to a low affinity of the o-ribosome pool for the host RBS which gives rise to a level of ‘interference’ as o-ribosomes translate the h-RFP. Increasing the ability of circuit genes to sequester ribosomes, for example by increasing RBS strength and expression level, abolishes the impact of this interference (Supplementary Fig. 5, discussed further in Supplementary Note 3). We do not observe translation of o-RFP by host ribosomes demonstrating that this appears to be a one-way interference (Supplementary Fig. 6). Increasing the size of the o-ribosome pool acts to dramatically increase o-RFP expression, demonstrating the utility of o-ribosomes to set a ‘circuit-specific protein budget’ (Fig. 1c, inset).

Use of o-ribosomes for gene expression has no effect on exponential growth rate (*p* = *NS* for pairwise comparisons at all IPTG levels), although there may be a slight effect on steady-state biomass (Supplementary Fig. 4). This is in agreement with previous observations using o-ribosome expression systems^15^, and with our model predictions. Our model demonstrates that due to interplay between metabolism, transcription and translation, the effect of o-ribosomes on host physiology is highly non-linear, which allows the cell to mitigate the impact of o-ribosome production to some extent (Supplementary Fig. 3, with a detailed description given in Supplementary Note 2.) Expression of o-RFP is on the order of tenfold smaller than that of the h-RFP. This may be due to inefficient o-rRNA production or inefficient o-ribosome assembly resulting in a smaller number of available ribosomes or due to the difference in strengths of the o-RBS (Supplementary Fig. 7, discussed further in Supplementary Note 4).

### Gene coupling in circuits utilising an o-ribosome pool

**Fig. 2:**
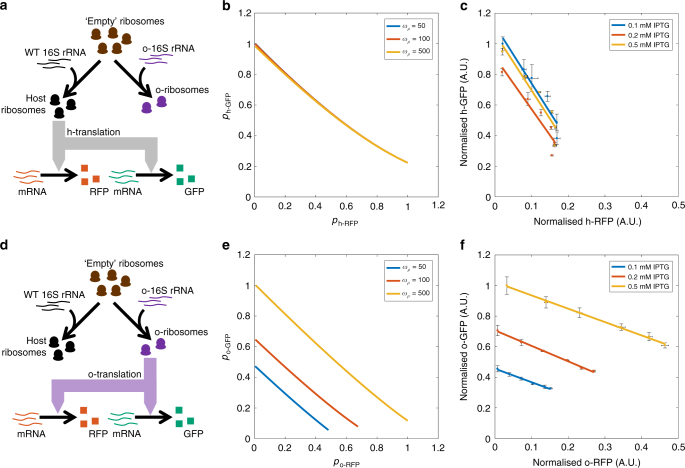
Gene coupling in gene circuits utilising either the host or orthogonal ribosome pool. Simulations of the steady-state concentrations of RFP and GFP normalised by the maximum protein production achieved across the o-rRNA transcription rates tested. *ω*_GFP_ = 100 and *ω*_RFP_ = 1 to 10^3^ mRNAs per min. o-rRNA production (*ω*_*ρ*_) was simulated at the RNAs per min as shown. Experimental data was produced by inducing RFP using AHL from 0 to 20 nM. Points are the mean steady-state fluorescence ± 1 SD normalised by maximum GFP expression obtained across different levels of IPTG treatment. *N* = 3. Raw data is shown in Supplementary Fig. 8 The isocost line is fit to the mean fluorescence as determined by FACS from cultures during mid-exponential growth (between 3 and 5 h post-induction dependent on the strain and circuit) and the gradient shown in Supplementary Fig. 9. o-ribosome production was induced using three different IPTG concentrations 0.1, 0.2 and 0.5 mM as shown. **a** Allocation of ribosomes in panels **b** and **c** where both circuit genes share the host ribosome pool. **b** Simulations of circuit using the host ribosome pool. **c** In vivo protein productions of circuit proteins using the host ribosome pool. FACS profiles shown in Supplementary Fig. 10. **d** Allocation of ribosomes in panels **e** and **f** where both circuit genes share the o-ribosome pool. **e** Simulations of the circuit using the o-ribosome pool for translation. **f** In vivo coupling observed in the circuit using the o-ribosome pool. FACS profiles are shown in Supplementary Fig. 11

Here we consider a new circuit consisting of two genes: the original RFP cassette and a new GFP cassette. GFP transcription was constitutively driven from the *P*_tet_ promoter and the host or o-ribosome pool utilised for translation, controlled by selection of RBS as described above. We determined the level of coupling between the two circuit genes by observing the slope of the isocost line of circuit gene expression during exponential growth; this quantifies the change in GFP as RFP is induced^3^ (Fig. 2). During balanced exponential growth, the concentration of RNAP and ribosomes is constant, creating a ‘fixed protein budget’. This is shared across the circuit genes so that as more is ‘spent’ on one gene, less can be ‘spent’ on another. This interaction is quantified in the slope of the isocost line^3^, which demonstrates the potential combinations in which the two proteins can be produced given the fixed budget. Gene coupling in the h-RFP, h-GFP circuit (utilising the host ribosome pool) results in a slope of −3.3; for every unit of RFP gained, ~3 units of GFP are lost (Fig. 2c). In this case the isocost line is nonlinear at maximum RFP expression; for consistency with previous studies^3^ and the following analysis we neglect this small non-linearity and favour fitting a straight line through all the points. Tuning the o-ribosome pool when utilising the host ribosomes as the translational resource has no effect on the coupling observed, consistent with our model predictions (Fig. 2b). Replacing the o-16S rRNA with its host counterpart allows overexpression of the endogenous 16S rRNA. This has no effect on gene coupling as measured by the isocost line slope (Supplementary Fig. 12, raw data shown in Supplementary Fig. 13). Replacing the host RBS sequences with the o-RBS to produce the o-RFP, o-GFP circuit and utilisation of the orthogonal translation system results in the coupling being reduced to 30% of that observed when using the host pool (Fig. 2f). Increasing IPTG increases gene expression but has negligible effect on coupling, as predicted by our model (Fig. 2e).

### Use of two ribosome pools reduces gene coupling

**Fig. 3:**
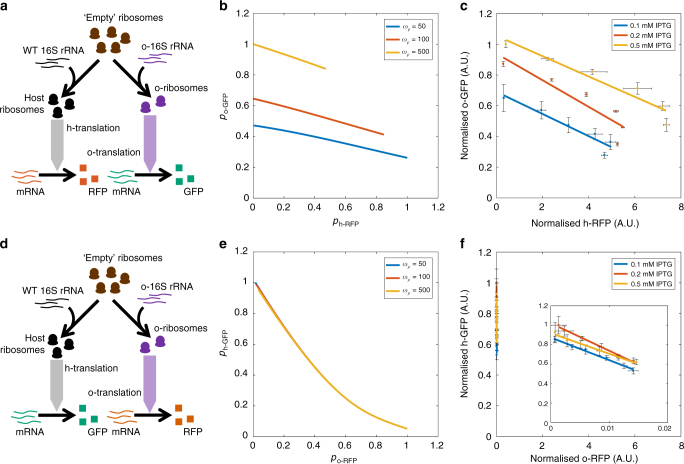
Gene coupling in circuits utilising different ribosome pools. Simulations of the steady-state concentrations of RFP and GFP normalised by the maximum protein production achieved across the o-rRNA transcription rates tested. *ω*_GFP_ = 100 and *ω*_RFP_ = 1 to 10^3^ mRNAs per min. o-rRNA production (*ω*_*ρ*_) was simulated at the rates shown. Experimental data was produced by inducing RFP using AHL from 0 to 20 nM. Points are mean steady-state fluorescence ± 1 SD normalised by maximum GFP expression obtained across different levels of IPTG treatment. *N* = 3. Raw data is shown in Supplementary Fig. 8. The isocost line is fit to the mean fluorescence as determined by FACS from cultures during mid-exponential growth (between 3 and 5 h post-induction dependent on the strain and circuit) and gradients calculated shown in Supplementary Fig. 9. o-ribosome production was induced using three different IPTG concentrations 0.1, 0.2 and 0.5 mM as shown. **a** Allocation of ribosomes in **b**, **c**. **b** Simulations of the circuit using host ribosomes for RFP expression and o-ribosome for GFP expression. **c** In vivo coupling of the h-RFP-o-GFP circuit. FACS profiles are shown in Supplementary Fig. 14. **d** Allocation of ribosomes in **e**, **f**. **e** Simulations of the circuit using the o-ribosome for RFP expression and host pool for GFP. **f** In vivo coupling of the o-RFP-h-GFP circuit. The inset shows the data on an expanded *x*-axis. FACS profiles are shown in Supplementary Fig. 15

Having successfully partitioned the ribosome pool, we tested the ability of these pools to act as a simple distribution mechanism for translational capacity. Maintaining the original circuit topology and function, we altered the RBS of each gene to create two new circuit variants: o-RFP, h-GFP and h-RFP, o-GFP. Our model predicts that placing the constitutively expressed GFP under control of the o-ribosome pool (h-RFP, o-GFP arrangement) acts to insulate the gene from competition with RFP and so significantly reduces gene coupling, over a range of o-ribosome pool sizes (Fig. 3a). Experimental validation of these predictions showed near complete abolition of the isocost line slope with coupling falling by over 95% (Fig. 3b). Varying IPTG levels shows this decoupling is highly robust, with IPTG acting only to tune expression (Fig. 3b).

The inverse arrangement, where the constitutively expressed GFP utilises the host ribosome pool and the induced RFP utilises the o-ribosome pool (the o-RFP, h-GFP circuit), results in increased gene coupling with the isocost line gradient increasing by over six times in comparison to coupling in circuits using the host ribosomes (Fig. 3c, f). Analysis of the model suggests that this is the result of competition for host ribosomal components, which are severely depleted. During translation of o-RFP, o-ribosomes are stabilised, which prevents release of ‘empty’ ribosomes, representing components of the ribosomal complex. This reduces the number of host ribosomes and so reduces host gene expression, including the expression of h-GFP (Supplementary Fig. 16, discussed further in Supplementary Note 5). These coupling observations are maintained if the two reporter genes are exchanged showing it is not a gene-dependent effect (Supplementary Fig. 17, raw data shown in Supplementary Fig. 18).

We extended this approach of using multiple ribosome pools in silico by simulating the use of one o-ribosome pool for each circuit gene (Supplementary Fig. 19, discussed further in Supplementary Note 6). Optimising the production of the two o-ribosome pools creates strong decoupling, with GFP falling less than 10% as RFP is induced. However, the RFP still shows a saturating response to increasing induction due to the finite o-ribosome pool size. There are also likely to be significant challenges in implementing multiple pools in vivo due to the cross talk found between different o-ribosome pools^15^.

Orthogonal ribosomes dissociate upon termination of translation into their small and large subunits. This creates competition for the large subunit between the host and orthogonal small subunits. By expressing a synthetic RNA containing both the (orthogonal) 16S and 23S rRNAs the two subunits can be permanently associated by an rRNA molecular tether. We extend our study by considering the use of tethered ribosomes in silico (Supplementary Fig. 20, discussed further in Supplementary Note 7). Our simulations demonstrate that the use of tethered ribosomes can increase protein production and that our previous observations of gene coupling are broadly maintained with the best decoupling achieved when the constitutive gene is translated by the tethered ribosome pool.

### Distributing resources increases violacein production

We have shown that by targeting the distribution of translational resources we can successfully reduce gene−gene couplings in simple circuits composed of fluorescent reporter genes. These genes do not have useful biological function beyond allowing visualisation. To demonstrate the utility of manipulating resource allocation in a biotechnological context, we consider the production of a metabolite from a multienzyme pathway.

As an example pathway we selected the well*-*characterised *vio* pathway from *Chromobacterium violaceum*^22^. This five-enzyme pathway produces violacein from l-tryptophan. Violacein has been shown to have anticancer and antibacterial properties, and has previously proved difficult to produce^22^. Additionally, the second enzyme in the pathway has previously been shown to induce a high cellular burden due to its large size and consequently large ribosome sequestration ability^6^. Due to violacein’s purple colour its production can be easily tracked. To demonstrate how resource-mediated competition can impact pathway function, we incorporated the violacein pathway into our model, as described in Supplementary Note 9. We divided expression of the pathway between two operons with the first enzyme of the pathway *vioA* placed under the control of *P*_tet_ and the other genes *vioB, C, D* and *E* in one polycistronic operon under the control of *P*_lux_, making expression inducible with AHL. For simplicity, we refer to this as the downstream cassette. In this arrangement, the flow of mass through the metabolic pathway mimics the information flow in an activation cascade which has previously been shown to be highly sensitive to the effects of resource competition^4^. We implemented this circuit in our model as described in Supplementary Note 9.

**Fig. 4:**
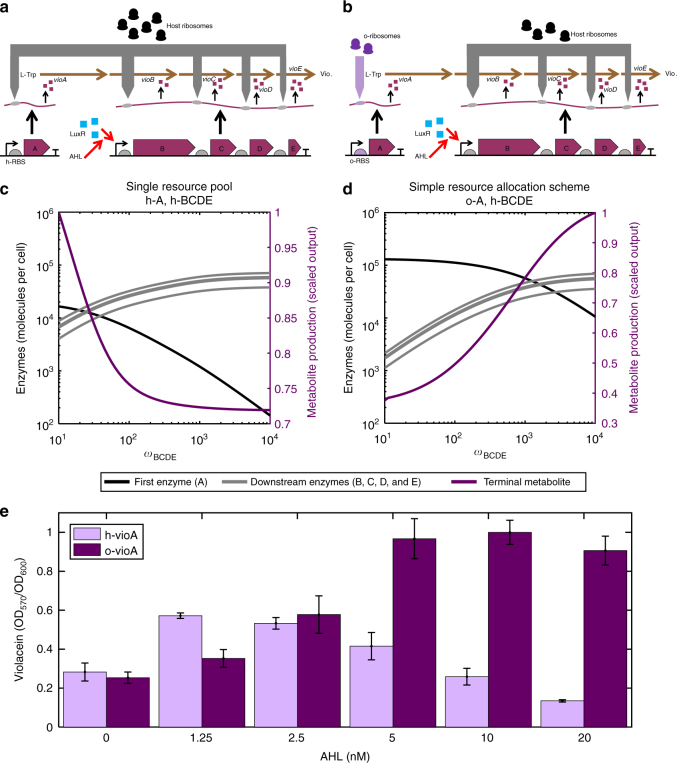
Resource allocation control increases production of violacein. Violacein is produce from l-tryptophan by a five-enzyme pathway. Experimentally the pathway is divided into two cassettes. *vioA* is constitutive expressed. The *vioBCDE* cassette is inducible under the control of AHL. Ribosomes are directed between the cassettes by use of ribosome binding sites as described previously. Simulations show the steady-state concentrations of the pathway enzymes and final metabolite. Note that the enzymes are divided by induction mechanism so that the downstream enzymes are depicted in the same colour. Variation in their levels is determined by protein size only. Scaled metabolite production represents the steady-state amount of the final metabolite in the pathway, scaled by the highest amount achieved across the induction. *ω*_*A*_ = 25 molecules per min. *ω*_*ρ*_ = 500 molecules per min. The downstream cassette is induced as shown by varying *ω*_*BCDE*_. These enzymes are translated by the host ribosome pool throughout. Other parameters are detailed in Supplementary Note 9. **a** Single resource pool. Use of host ribosomes by all genes. **b** Simple resource allocation scheme. *vioA* translation by the orthogonal ribosome system. **c** Simulation of the cassettes sharing a single pool of resources. Enzyme A is expressed utilising the host system. **d** Simple resource allocation scheme by allocating enzyme A to the orthogonal ribosome pool. **e** Violacein production in vivo per cell at 24 h post induction. Normalised by the largest production per cell achieved across all conditions. Bars represent means ± 1 SD. *N* = 3

In the absence of resource competition, it would be expected that increasing AHL would increase the pathway flux due to increased expression of the pathway enzymes. However, when a single pool of ribosomes is used for the expression of both cassettes, our model predicts that competition emerges (illustrated in Fig. 4a). As the downstream cassette is induced, the first constitutively expressed enzyme falls (Fig. 4c). This results in a concurrent decrease in metabolite production. We have shown above that the expression of a constitutive gene can be maintained if this gene is targeted to the orthogonal ribosome pool. This observation extends to complex pathways, with the first enzyme’s decrease in response to the induction of the downstream cassette being significantly reduced in comparison to using only one ribosome pool (Fig. 4d). This change in enzyme distribution results in a concurrent increase in metabolite production across the ranges of induction simulated. It should be noted that metabolite production does begin to plateau at high induction. Our simulations predict that the use of this resource allocation scheme can improve final metabolite production significantly.

To validate these predictions, we implemented the *vioABCDE* pathway as described above in *E. coli*. We monitored cell growth and violacein production for the cases where (i) *vioA* is produced from the host pool and (ii) *vioA* is produced from the o-ribosome pool. A representative plate showing violacein accumulation is shown in Supplementary Fig. 21. When using the host ribosome pool for expression of all of the pathway components, we see a peak of production of violacein at intermediate concentrations of AHL, with both low and high concentrations of AHL yielding poor production, as expected from the predicted couplings deriving from ribosomal competition (Fig. 4e). Reducing the cost of protein production by using the orthogonal ribosome system allows for higher production yields per cell that increase monotonically with AHL concentration (Fig. 4e). Despite these higher yields we do not observe any significant decrease in growth rate (Supplementary Fig. 22). Moreover, we do not observe the emergence of significant numbers of mutants which do not produce violacein in either of the strains tested, suggesting the decrease in production yields is due to the competition for translational resources between different segments of the pathway (Supplementary Fig. 23).

### Design of a dynamic resource allocation controller

**Fig. 5:**
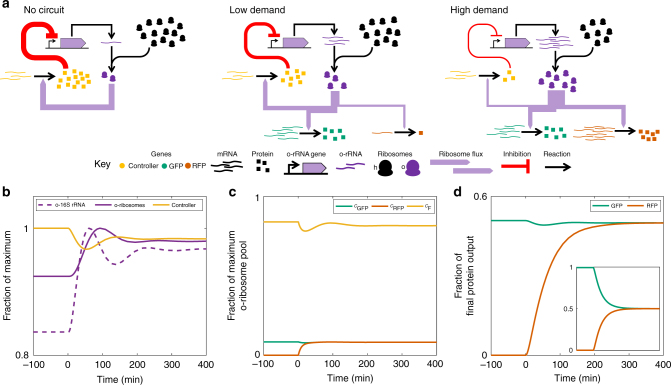
Operation of the negative feedback controller. **a** Structure and function of the negative feedback controller. In the absence of the circuit genes, repression of o-rRNA production is high, and so co-option of ribosomes to the orthogonal pool is low. Upon the introduction of a low demand circuit the o-ribosome pool is redistributed to express both circuit and controller genes. This reduces translation of the constitutively expressed controller due to resource competition, and as a result o-rRNA transcription increases. As circuit demand increases (here RFP transcription increases), increased resource competition results in a greater fall in controller translation. This reduces the repression of the o-rRNA production, allowing more co-option of ribosomes from the host ribosome pool. **b** Simulation showing the changing concentrations of the controller components in response to induction of RFP, which creates increased ribosomal demand. Note the narrow *y*-axis range. **c** Changing distribution of the translation complexes, *c*, in response to RFP induction of genes utilising o-ribosomes for translation. *c*_*F*_, controller mRNA-o-ribosome translation complex. **d** Protein output over time. Inset, coupling in the o-ribosome pool without controller. The induction rate of the o-rRNA was tuned so that both models produce the same protein concentration

Although we have shown that using separate host and o-ribosome pools can significantly reduce coupling between co-expressed genes, finite resource limitations still result in a saturated input−output response profile, while using a single orthogonal pool results in significant resource-mediated coupling. This coupling can, however, be exploited to design a feedback controller which acts to dynamically increase the circuit translational capability in line with increasing demand, thus alleviating both resource-mediated coupling and saturation effects due to a fixed allocation of orthogonal ribosomes (Fig. 5). Increasing ribosome biosynthesis is not experimentally tractable so our controller acts to change the ratio of orthogonal to host ribosomes. We exploit the constitutive production of a repressor which utilises the o-ribosome pool for its own translation and inhibits the expression of the o-16S rRNA. Constitutive production of the repressor mRNA means that repressor protein levels act as a sensor for translational demand (Fig. 5). To guide our design, we first implemented the feedback mechanism in our model (see Supplementary Note 1). We demonstrate the function of our controller by considering the constitutive expression of one gene and simulating its response to the stepped induction of a second gene (Fig. 5). When circuit demand is low, before the second gene induction, competition between the circuit and the controller is low (Fig. 5b, note *y*-axis of b). This results in high expression of the controller and therefore high repression of the o-rRNA, meaning that few ribosomes are co-opted from the host. Upon induction of the second gene, the demand for o-ribosomes increases (Fig. 5b). The repressor mRNAs will remain largely unaffected, but their translation falls due to increased competition (Fig. 5b). The decrease in repressor production results in relief of the inhibition of the o-16S rRNA gene and so increased o-rRNA production and increased co-option of host ribosomes (Fig. 5a). This results in the maintenance of circuit protein production as other circuit genes are induced (Fig. 5c). Note that saturation effects cannot be abolished entirely as eventually other cellular components will become limiting, such as RNA polymerase or tRNAs. Although a simple representation of microbial physiology, our model’s metabolism is able to provide sufficient energy to drive translation at high levels of protein production (Supplementary Fig. 24). This suggests that it is the distribution of proteins, including the number of free ribosomes, which results in saturation in this instance.

**Fig. 6:**
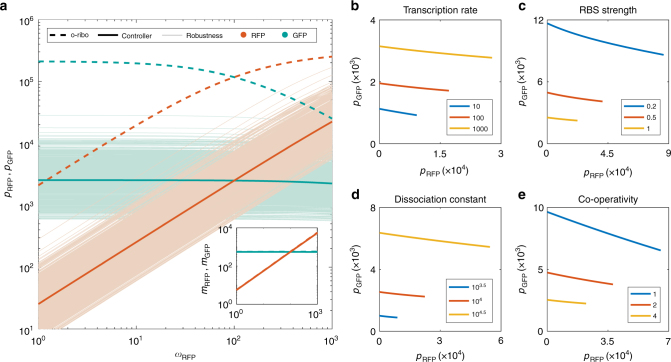
Design of the feedback controller. **a** Function and robustness of the controller decoupling in comparison to using the o-ribosome pool alone. The optimal controller parameters were perturbed by drawing random values (*N* = 1000) between ±50% of the original value. All parameters controlling o-rRNA and controller protein were allowed to vary. Inset, mRNA levels of RFP and GFP. Legend explanation: o-ribo, uncontrolled o-ribosome pool (i.e. when *ω*_*F*_ = 0 mRNAs per min); Controller, optimal feedback controller; Robustness, action of the perturbed controllers. **b** Impact of varying the maximal transcription rate of the o-rRNA. **c** Impact of varying the strength of the RBS **d** Impact of varying the dissociation constant of the repressor. **e** Impact of varying the Hill function coefficient of the repressor

To test the robustness of our controller design to likely levels of uncertainty and variability arising from potential experimental implementations, we carried out sensitivity and robustness analyses around the optimal solution identified (Fig. 6). This indicated that the feedback topology is highly robust, with all designs tested showing a better mRNA-protein mapping than the circuit using the o-ribosome pool without control, including those parameters which are difficult to design such as the ‘transcriptional energy threshold’ *o*_*ρ*_ (Fig. 6a, inset). However, we do see the emergence of a trade-off between decoupling ability and gene expression, with decoupling coming at a cost to gene expression (Supplementary Fig. 25). Our sensitivity analysis shows that the controller needs to have a high ability to sequester o-ribosomes. Increasing the o-rRNA transcription rate and  the controller's RBS strength is predicted to increase the ability of the controller to decouple genes (Fig. 6b, c), with the former also raising gene expression while the latter reduces it. Varying the dissociation constant across a tenfold range allows the protein levels to be tuned at no cost to the level of decoupling achieved (Fig. 6d), while varying the Hill function coefficient shows the need for the controller to be highly non-linear (Fig. 6e).

### The controller decouples co-expressed genes in vivo

**Fig. 7:**
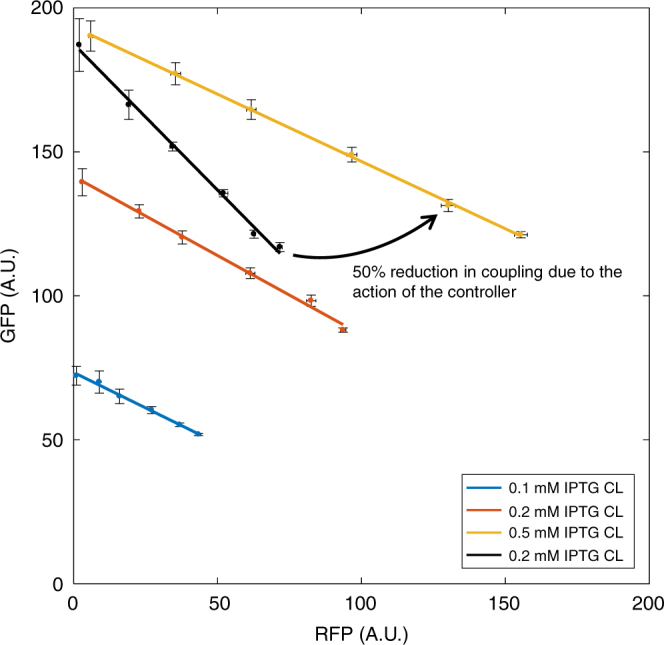
In vivo implementation of the prototype controller. Response of constitutively expressed o-GFP as o-RFP is induced. o-RFP was induced using AHL from 0 to 20 nM. Points are the mean steady-state fluorescence ± 1 SD as determined by FACS from cultures during mid-exponential growth (between 3 and 5 h post-induction dependent on the strain and circuit). *N* = 3. The level of gene expression from the controller can be tuned with IPTG as shown (CL, closed loop). For comparison the o-RFP, o-GFP results which produce comparable initial GFP levels are also shown (OL, open loop). FACS profiles are shown in Supplementary Fig. 26. All OL and CL data is shown in Supplementary Fig. 27. Isocost lines are fit to mean values as described in the ‘Methods’ section and gradients shown in Supplementary Fig. 9

Having demonstrated the feasibility of using a feedback controller to dynamically allocate ribosomes between host and circuit, we implemented a prototype controller in vivo. Using the results of our sensitivity analysis as a guide, we based our controller on the strongly binding LacI repressor (*k*_*D*_ ≈ 0.02 nM^23^), which also shows a highly non-linear mode of action due to the dimerisation steps required to produce the functional complex (Fig. 6). We selected the strong *P*_lacI_^q^ promoter to drive LacI transcription^24^, and used the same orthogonal RBS as previously. We deleted the host chromosomal *lacI* gene and placed the exogenous copy with promoter and o-RBS into the plasmid with the o-16S rRNA. Comparing the 0.5 mM IPTG treatment of the controlled circuit with the uncontrolled o-RFP, o-GFP circuit of equivalent initial GFP number (i.e. equivalent demand for ribosomes), we found that the controller decreases coupling by 50% (Fig. 7). Tuning the controller threshold with IPTG allows the tuning of protein levels at no cost to decoupling (consistent with Fig. 6d).

## Discussion

To control cellular processes synthetic biologists and biotechnologists often use regulation of gene expression; by regulating transcription we assume protein levels will follow. However the use of a common pool of cellular resources results in the emergence of hidden interactions, ‘couplings’, between genes which are not immediately apparent from circuit topologies. This can result in a breakdown in the relationship between transcriptional regulation (input) and protein levels (output). In this paper, we have shown the feasibility of dividing the cell’s translational resources to reverse this breakdown.

We have demonstrated that the use of orthogonal ribosomes is non-toxic to host cells (in agreement with previous reports), and that they can be used successfully for the targeted expression of synthetic circuit genes. We have shown that manipulating the size of the orthogonal ribosome pool provides an additional dial for controlling gene expression^25^. Our system shows a degree of interference between the two ribosome pools, with orthogonal ribosomes having a propensity to translate mRNA that is targeted to the host pool. This has potentially significant effects in biotechnological processes if the mRNA’s protein product is toxic (such as toxin−antitoxin pairs). However, we find that this interference effect is abolished in high demand circuits, and we also note that there is no interference caused by host ribosomes translating orthogonal genes, which suggests that toxic components can be safely expressed from mRNAs with an orthogonal RBS.

At present, the orthogonal translation system yields tenfold lower protein expression than when using the host pool alone, with a corresponding increase in gene expression noise. This could have significant implications for potential biotechnological applications which require high uniform expression. However, we have demonstrated that the o-ribosome system is sufficiently efficient to allow for increased production of a highly bioactive molecule, violacein, which has antimicrobial and anticancer properties. It has also recently been shown that yields of violacein produced from enzymes translated by an orthogonal translation system were sufficient for biotechnological applications^19^. Increasing protein expression in the o-ribosome system could be achieved via a number of independent and complementary approaches: (1) re-engineering the o-RBS using thermodynamic models to increase binding efficiency^17^, (2) using tethered ribosomes where the large and small subunits are linked by a linker RNA as briefly discussed above^26^, (3) directed evolution or modification of the host to improve ribosome biosynthesis. Numerous studies have identified pathways which appear to limit protein synthesis or ribosome biogenesis, and rational re-design of these would also feasibly lead to increased o-ribosome production^27–29^.

We have shown that by carefully selecting which genetic modules utilise either the host or orthogonal ribosome pools we can reduce hidden interactions between co-expressed genes, including in a complex multienzyme pathway. Our simple model of cellular physiology is sufficient to determine which modules should utilise the o-ribosome pool to achieve the best coupling reduction. The greatest level of decoupling is achieved when constitutively expressed genes utilise the orthogonal pool while dynamically regulated genes utilise the host pool. In this manner, the orthogonal pool appears to act as an insulation device preventing changes in the host ribosome pool being transmitted to the constitutively expressed gene.

When all circuit genes are translated by the orthogonal ribosome pool, resource-mediated competition between circuit genes emerges due to the fixed ribosomal budget allocated to that pool, resulting in an effective autoinhibition of each gene with increasing induction. We showed that dynamic allocation of the orthogonal ribosomal budget can be used to mitigate this saturating effect. Taking inspiration from control theory, we took advantage of gene coupling to design a negative feedback controller which acts to dynamically divert resources to the synthetic circuit as demand for them increases. This controller thus acts to alleviate both ribosome-mediated saturation and gene−gene coupling. Guided by the analysis of our model, we implemented a prototype controller, based on the repressor LacI, and found that it could reduce gene−gene coupling in a simple two-gene circuit by 50%. The decoupling ability of the controller is robust, with only protein levels, not coupling, being tuned by the addition of IPTG. Although our model predicts that coupling can be reduced further than the 50% observed in our prototype design, the remaining uncontrolled coupling observed experimentally is likely to be due to factors such as transcriptional competition between genes for RNA polymerase (which is only approximated in our model). Significant further work is needed to develop mechanistic modelling frameworks and design rules to enable the construction of optimised controllers for more complex circuits and pathways.

In previous studies, a number of *transcriptional* resource allocators have been developed, either by directing the core polymerase via use of orthogonal *σ* factors (analogous to our use of multiple ribosome pools), or by using feedback to control RNA polymerase expression across species^30, 31^. In another approach, Venturelli et al.^32^ proposed and implemented a global resource allocator based on increasing decay rates of host mRNAs to reduce ribosomal competition. Our dynamic resource allocator complements these efforts by providing another potential layer of control at the translational level, which multiple studies have shown is the key limiting factor in determining bacterial gene expression. Our results show that translational capacity can be controlled in order to optimally manage the consumption of host resources and reduce circuit context dependency. These decoupling mechanisms provide designers with multiple means to reduce host−circuit interactions and improve circuit modularity, without the need for costly and time-consuming rounds of experimentation and library screening, thus facilitating the real-world implementation of synthetic gene circuits in future biotechnological applications.

## Methods

### Mathematical modelling

The full model and modifications are described in detail in Supplementary Note 1. All ordinary differential equation models were simulated to steady state using custom software written in MATLAB 2016a (The MathWorks Inc., Natick, MA, USA). Numerical methods are detailed in Supplementary Note 1. Parameters are detailed in Supplementary Tables 1 and 2.

### Cloning procedures and construction of reporter strains

The characteristics of the bacteria, plasmids and primers used in this study are described in Supplementary Tables 3 and 4. DNA manipulation was carried out following standard protocols^33^. Plasmid DNA was isolated from bacterial cells using a commercial QIAprep Spin Miniprep Kit (Qiagen, Hilden, Germany). Restriction endonucleases were purchased from New England Biolabs (NEB, Ipswich, MA, USA). Plasmid maps of those plasmids produced in this study are shown in Supplementary Fig. 28.

Plasmid pSEVA63-Dual was derived from MBP 1.0, the original circuit containing the genes for constitutive GFP and inducible RFP expression^3^. The circuit was amplified using the pair of primers Dual F/-R, digested with *Pac*I/*Sac*I and cloned into pSEVA631 using the same restriction endonucleases^34^.

Two subsequent cloning steps were carried out to replace the original RBS sequence of RFP in the plasmid. First, the original RBS of RFP was replaced with the orthogonal sequence (ACAATTTTCATATCCCTCCGCAA) using the Fast Cloning method^35^ with primers o-RFP F/-R resulting in the plasmid pEMG-o-RFP-h-GFP. The RBS of GFP was replaced in this circuit using the pair of primers Dual F and Dual R and the PCR product was cloned into pSEVA631 as described above, which resulted in the plasmid pSEVA63-o-RFP-h-GFP. Plasmids pSEVA63-h-RFP-o-GFP and pSEVA63-o-RFP-o-GFP were generated with a similar approach in this case using primers o-GFP F/-R.

The RBS of the *lacI* gene present in the plasmid pRSF ribo-Q1 O-*gst-cam*^21^ was replaced with the orthogonal version also following this strategy: four partial fragments representing the whole plasmid were amplified by PCR with the respective primer pairs (pAS2 o-lacI pt F/-R, pAS2 o-lacI pt2 F/-R, pAS2 o-lacI pt3 F/-R and pAS2 o-lacI pt4 F/-R). Each product had overlapping regions and one of these regions contained the orthogonal RBS directly upstream of the *lacI* gene. DpnI treatment was carried out to remove the template DNA after the PCR reaction, and the DNA fragments were ligated by isothermal assembly^36^ yielding plasmid pRSF ribo-Q1 o-gst-cam o-lacI.

For the construction of the control plasmid in which the two fluorescent reporters are swapped (pSEVA63sw-o-GFP-h-RFP), four DNA fragments corresponding to the plasmid backbone, the *gfp* gene containing the o-RBS, the *luxR* gene and the *rfp* gene were amplified using the plasmid pSEVA63Dual as a template and their corresponding primer pairs (63sw F/-R, GFPsw F/ oGFPsw R, oGluxRsw F/ luxRsw R, RFPsw F/-R). As before, all amplicons were treated with DpnI and ligated by isothermal assembly. In a similar approach, the plasmid pSEVA63sw-h-GFP-o-RFP was obtained with following primer pairs: 63sw F/-R, GFPsw F/-R, luxRsw F/ oRluxRsw R, oRFPsw F/RFP sw R.

The MG1655ΔlacIZYA strain—this is the parental MG1655 strain lacking the *lacI* gene and the *lac* operon (i.e. the *lacZYA* genes)—was constructed by replacing the target genomic regions with a kanamycin antibiotic cassette (pKD4) using primers lac KO F/-R, followed by removal of the kanamycin resistance using the FLP recombinase using a previously described method^37, 38^. Genomic deletions were confirmed by PCR and by ensuring that the activity of β-galactosidase was not present in the strain.

All plasmid constructs described above were introduced into either *E. coli* DH5*α* or DH5*αλpir* strains by transformation for DNA amplification. All experiments in this paper were performed in *E. coli* MG1655 after transformation with the corresponding plasmids with the exception of those involving the feedback controller (pRSF ribo-Q1 o-gst-cam o-lacI), which were carried out in MG1655ΔlacIZYA to prevent cross talk between the endogenous *lacI* gene and the controller.

### Violacein pathway assembly

The violacein biosynthetic pathway used for the study of competition is composed by a *vioA* gene constitutively expressed and the *vioBCDE* operon under control of LuxR. All fragments (three in total) were chemically synthesised (GENEWIZ, South Plainfield, NJ, USA) and edited as follows: the original RBS in each of the *vio* genes from *C. violaceum* ATCC 12472 was replaced with a strong RBS (BBa_B0034); the *Sac*I restriction site present in *vioC* was removed without affecting the resulting amino acid sequence, during the synthesis; an *Avr*II restriction site was introduced between *luxR* and *vioA*. The resulting fragments containing homology regions were then inserted into the plasmid pSEVA631 previously digested with *Pac*I and *Sac*I using isothermal assembly yielding plasmid pSEVA63-Hvio.

In a following step, the RBS of *vioA* was replaced with the o-RBS. To this end, we generated two DNA fragments by PCR: one containing the *vioA* gene with the o-RBS (primers ovioA F/-R), and another containing the 553 bp upstream the *vioA* gene finishing with the o-RBS in the 3′ end (primers lux-vioA F/-R). These two segments were joined by SOEing PCR^39^. The resulting PCR product was cloned into the pSEVA63-Hvio by restriction digestion (with *Avr*II and *Sac*I) and ligation resulting in the plasmid pSEVA63-Ovio.

### Cell culturing for coupling experiments

*E. coli* strains used in this study were always grown in Luria-Bertani (LB) medium at 37 °C. The antibiotics kanamycin (50 μg mL^−1^), ampicillin (150 μg mL^−1^) and gentamicin (20 μg mL^−1^) were added when necessary. In a typical experiment, for each of the biological replicates, a single colony from the strain of interest was taken from a fresh plate and cultured overnight in 1 mL of liquid medium in 24-well plates (500 rpm, PMS-1000i Microplate Shaker, Grant, Shepreth, UK). These cultures were diluted 500-fold in the same medium and further grown in the same conditions. Once they reached the exponential phase (OD_600_ = 0.2–0.3), 2 μL of the culture were transferred into 1 mL of fresh medium containing IPTG (Sigma-Aldrich, St. Louis, MO, USA, final concentrations of 0.1, 0.2 and 0.5 mM) and/or *N*-acyl homoserine lactone (AHL, Sigma-Aldrich, St. Louis, MO, USA, final concentrations of 1.25, 2.5, 5, 10, 20 nM) and cultured in the same way as in the previous two steps.

### Fluorescence measurements

Growth and population-level fluorescence of these cultures was monitored over time as follows: every hour the OD_600_ of the cultures was determined using a CLARIOstar microplate reader (BMG Labtech, Ortenberg, Germany). At the same time single cell fluorescence measurements were collected as follows: 50 μL aliquots were taken from each well and mixed with 150 μL of PBS. The volume of the culture in the wells was kept constant by replenishing with the same volume of fresh medium including the same concentration of the inducers. The suspension of cells in PBS was loaded into an Attune NxT Flow Cytometer (ThermoFisher, Waltham, MA, USA) and analysed for GFP and RFP expression using blue (excitation 561 nm; emission 620/15 nm) and yellow (excitation 561 nm; emission 620/15 nm) lasers respectively. For each sample, 20,000 events were analysed and populations means were estimated using the default software of the instrument.

### Quantification of gene coupling

Coupling between genes was determined by fitting isocost lines as described in ref. ^3^. Briefly, the mean steady-state protein production, as measured by fluorescence, was determined as above. The constitutive gene (GFP) was plotted against the induced gene (RFP) and a straight line fit through all the points. The gradient forms a measure of the gene−gene coupling.

### Growth rate determination

For initial characterisation of o-ribosome burden cells were cultured as outlined above. Two microliters of culture was transferred into 1 mL fresh medium at the IPTG concentration stated. Growth as OD_600_ was measured using a CLARIOstar microplate reader over 7 h. Growth rate was determined by taking natural logarithms of OD_600_ and fitting a straight line to the linear portion of the graph.

### Characterisation of violacein-producing strains

The strains producing violacein were inoculated into liquid LB medium supplemented with tryptophan (1 g L^−1^) and the appropriate antibiotics, and grown overnight at 37 °C and 160 rpm. After this, the cultures were diluted 250-fold in 50 mL of the same medium containing both 0.2 mM IPTG and AHL (final concentration 1.25, 2.5, 5, 10, 20 nM) and cultured in 250 mL volume of flasks as above. Every 2 h 1 mL of the culture from each flask was centrifuged (17,000×*g*, 10 min) and the pellet was resuspended in absolute ethanol. Violacein was extracted by incubating the cell suspension at 95 °C for 10 min followed by pelleting cell debris (17,000×*g*, 10 min). The violacein present in the supernatant was determined spectrophotometrically at 570 nm (Evolution 60S UV-Visible Spectrophotometer, ThermoFisher, Waltham, MA, USA). The insoluble cell debris remaining after the extraction was resuspended in water and its optical density determined at 600 nm.

### Data availability

All equations and parameter values used in the computational model are available in the Supplementary Notes. Example of MATLAB code is available online at https://github.com/apsduk/Nat-Commun-2018. Experimental data is available upon request.

## Electronic supplementary material

Supplementary Information
