# Supplementary material for: Dynamic allocation of orthogonal ribosomes facilitates uncoupling of co-expressed genes
Source: Nat Commun. 2018 Feb 15;9:695. doi: 10.1038/s41467-018-02898-6 (PMC5814443; doi:10.1038/s41467-018-02898-6)
Supplement: Supplementary file 1 — Supplementary Information [file 41467_2018_2898_MOESM1_ESM.pdf]

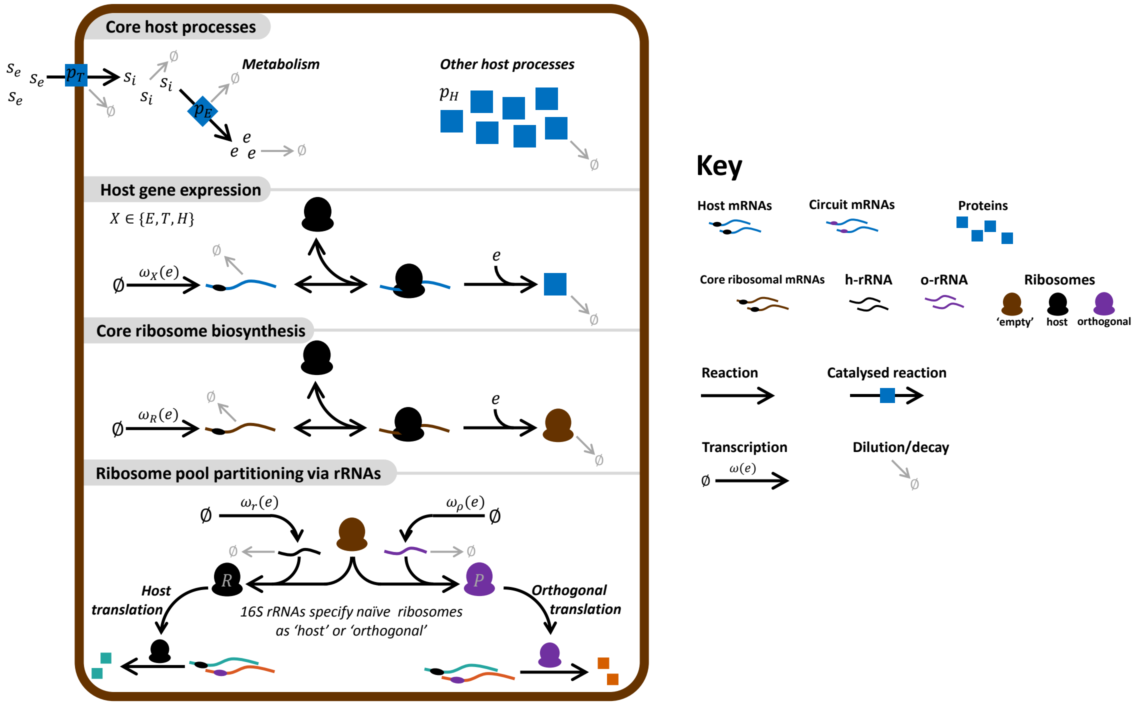

Supplementary Figure 1: **Model schematic** Main host processes captured by our model are metabolism, gene expression and ribosome biosynthesis. *NB:* Not all reactions are shown to simplify the schematic. All reactions are described in Supplementary Note 1.

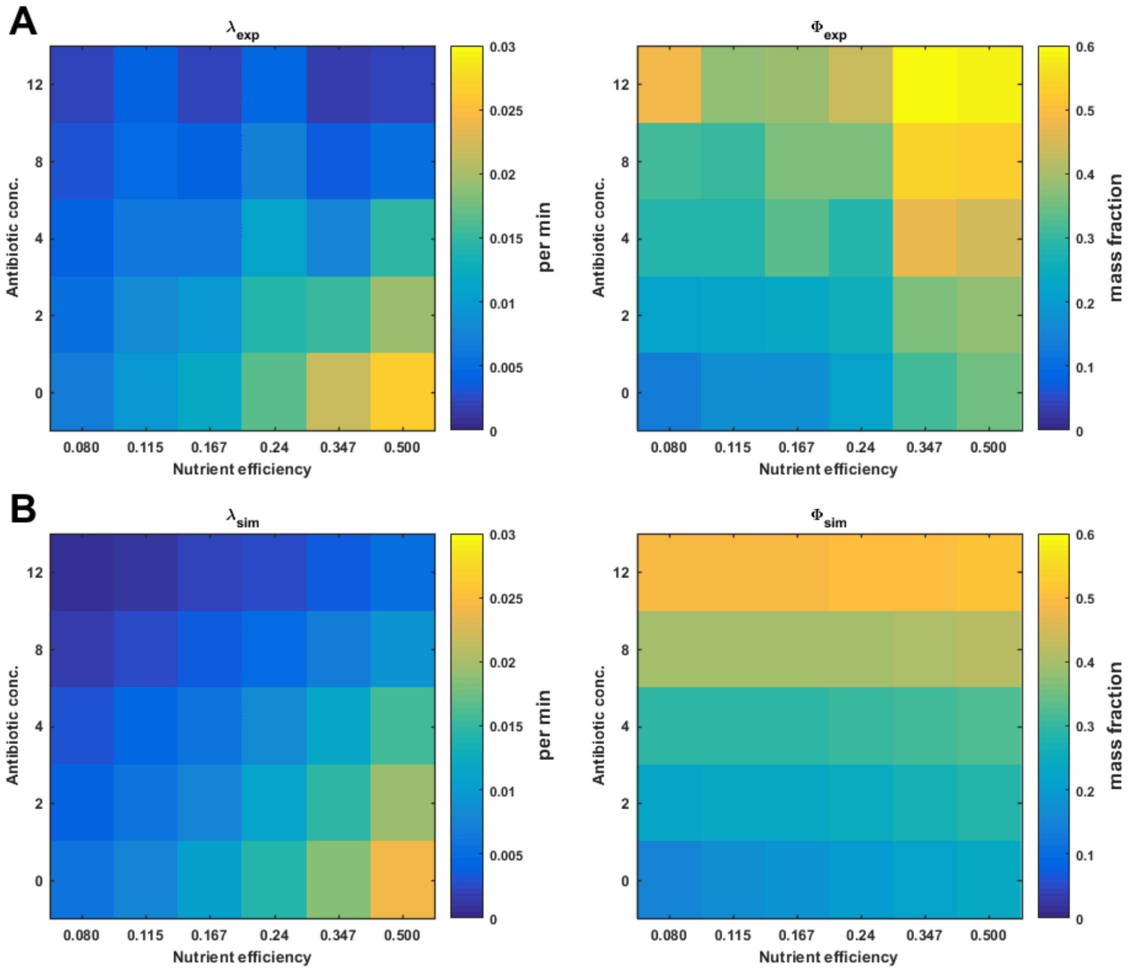

Supplementary Figure 2: **Comparison of host model with Scott *et al.* experimental data. (A)** Experimental results produced by Scott *et al.* Data were produced by growing cells at different nutrient qualities (which are quantified here as nutrient efficiency, s_s) and ribosome-toxic antibiotic concentrations. Proteins and RNA were harvested and used to calculate ribosomal mass fraction (see [1] for details). **(B)** Simulations of the growth rate and ribosomal mass fraction.

Parameter	Value	Units	Notes	Ref
s_E	10^4	mc	External substrate	[2]
φ_e	0.5	–	Nutrient efficiency	[2]
v_T	728	mc·min^{-1}	Maximal nutrient import	[2]
v_E	5800	mc·min^{-1}	Maximal substrate-to-energy conversion	[2]
k_T	1000	mc	Transporter Michaelis-Menten constant	[2]
k_E	1000	mc	Enzyme Michaelis-Menten constant	[2]
$\omega_{\{T,E\}}$	4.14	mc·min^{-1}	Maximal transport and enzyme transcription rate	[2]
ω_H	948.93	mc·min^{-1}	Maximal general host protein transcription rate	[2]
ω_R	930	mc·min^{-1}	Maximal ribosomal mRNA transcription rate	[2]
ω_r	3170	mc·min^{-1}	Maximal rRNA transcription rate	$*$
$o_{\{T,E,H\}}$	4.38	mc	Host genes transcription energy threshold	[2]
$o_{\{R,r\}}$	426.87	mc	Ribosomal genes transcription energy threshold	[2],$*$
$b_{\{T,E,H,R\}}$	1	mc^{-1}·min^{-1}	mRNA-ribosome binding rate	[2]
$u_{\{T,E,H,R\}}$	1	min^{-1}	mRNA-ribosome unbinding rate	[2]
b_r	1	mc^{-1}·min^{-1}	rRNA-empty ribosome binding rate	$*$
u_r	1	min^{-1}	rRNA-empty ribosome unbinding rate	$*$
$\delta_{m_{\{T,E,H,R\}}}$	0.1	min^{-1}	Host mRNA degradation rate	[2]
δ_r	0.1	min^{-1}	rRNA degradation rate	$*$
$\delta_{p_{\{T,E,H\}}}$	0	min^{-1}	Protein degradation rate	[2]
δ_{p_R}	0	min^{-1}	Ribosome degradation rate	[2]
$n_{\{T,E,H\}}$	300	aa	Average *E. coli* gene length	[2]
n_R	7459	aa	Ribosomal protein component length	[2]
k_H	152219	mc	Host protein transcription threshold	[2]
h_H	4	–	Host protein transcription Hill constant	[2]
γ_{max}	1260	aa·min^{-1}·$(e$ mc$)^{-1}$	Maximal elongation rate	[2]
k_γ	7	$(e$ mc$)$	Elongation energy threshold	[2]
M	10^8	aa	Size of proteome	[2]

Supplementary Table 1: **Model parameters** Most model rate constants were derived in [2] by fitting to the data in [1]. We assume that the ribosomal protein complex (denoted R) has the same parameters as the ribosome as a whole in [2]. We assume that the transcriptional threshold o_r is the same as for the protein component. We assume that the rRNA has the same stability as host mRNAs (i.e. $\delta_r = \delta_{m_X}$) and that the binding/unbinding rates are diffusion limited (i.e. $b_r = u_r = 1$). We fit ω_r to the data in [1]. $*$, Fit in this study. Units: aa, amino acids, mc, number of molecules.

Parameter	Value	Units	Rational
ω_Y	Varied	mc·min^{-1}	Varied between biologically feasible values. 0 and 10^3 [2]
o_Y	4.38	mc	Assumed to be the same as the host genes
b_Y	1	mc^{-1}·min^{-1}	Assumed to strong ≈ 1 see Section Supplementary Note 3
u_Y	1	min^{-1}	
n_Y	300	aa	Average length of *E. coli* protein
δ_{m_Y}	0.1	min^{-1}	Assumed to be the same as the host's mRNAs
δ_{p_Y}	0	min^{-1}	Assumed dilution only
ω_ρ	Varied	mc·min^{-1}	Varied between biologically feasible values. 0 and 10^3 [2]
ω_ρ	350	mc·min^{-1}	In the controlled system, $*$
o_ρ	4.38	mc	Assumed to be the same as the host genes
b_ρ	1	mc^{-1}·min^{-1}	Assume same as host rRNA
u_ρ	1	min^{-1}	Assume same as host rRNA
δ_ρ	0.1	min^{-1}	Assume same as host rRNA
ω_F	1000	mc·min^{-1}	$*$
o_F	4.38	mc	Assumed to be the same as the host's genes
b_F	1	mc^{-1}·min^{-1}	$*$
u_F	1	min^{-1}	
n_F	300	aa	Average length of *E. coli* protein
δ_{m_F}	0.1	min^{-1}	Assumed to be the same as the hosts
δ_{p_F}	0	min^{-1}	Assumed dilution only
k_F	10^4	mc	$*$
h_F	4	–	$*$

Supplementary Table 2: **Parameter values for circuit gene, o-ribosome and controller production** $*$, determined in the optimisation routine. Units: aa, amino acids, mc, number of molecules.

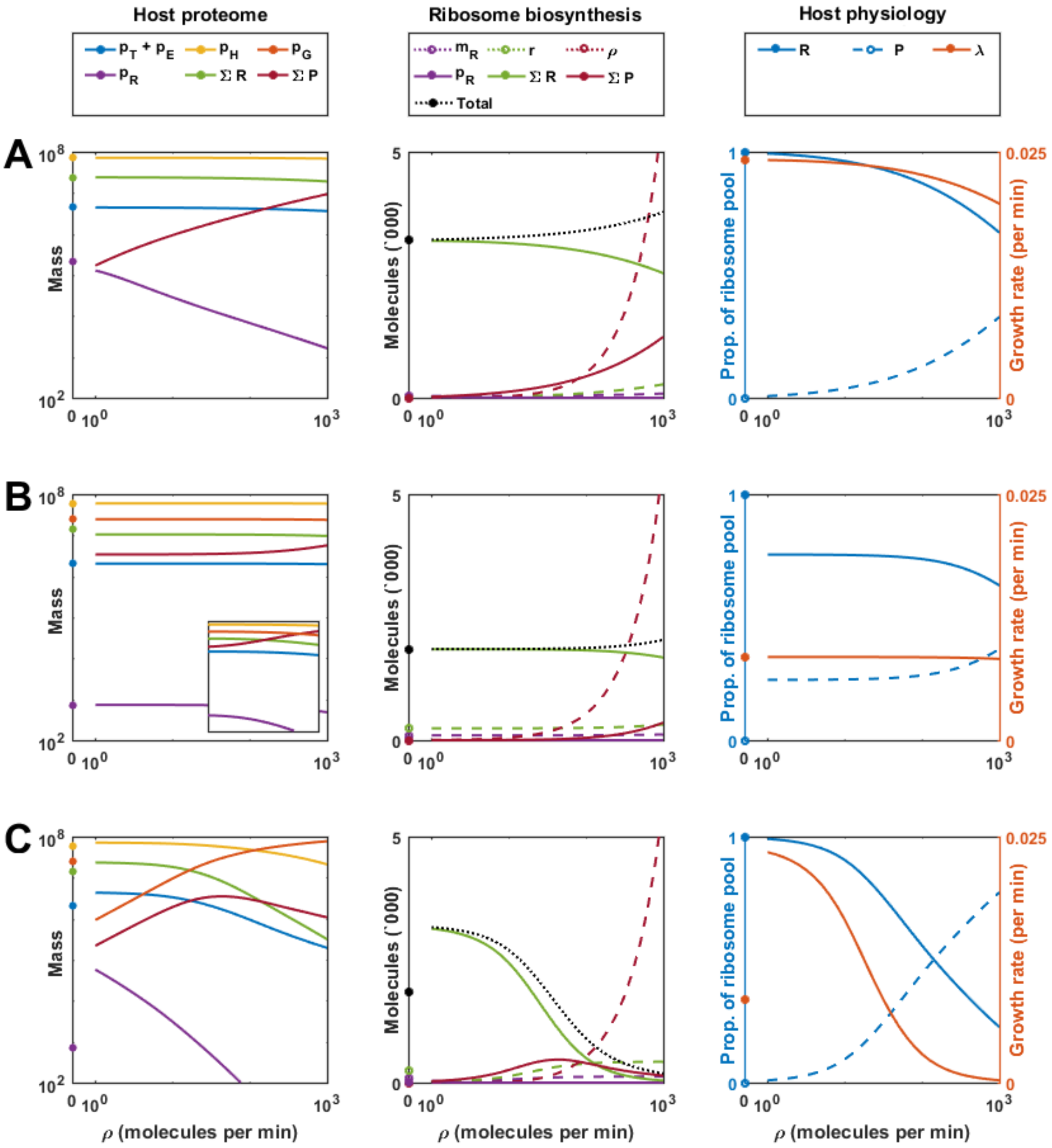

Supplementary Figure 3: **Simulations of the impact of orthogonal ribosomes on host physiology.** The impact of orthogonal ribosome production was assessed by simulating the response to increasing o-rRNA transcription rate (ω_ρ). Single markers show the results when using the host ribosome pool in the absence of orthogonal ribosome production. Host effects are assessed by observing changes in the proteome, ribosome biosynthesis and macroscopic effects such as ribosome distribution and growth rate. See Supplementary Note 2 for further discussion. Legend explanation: $p_T + p_E$, metabolic enzymes; p_H, host proteins; p_G, reporter protein; p_R, 'empty' ribosome; ΣR, free and translating host ribosomes; ΣP, free and translating o-ribosomes; m_R, mRNA of the ribosomal protein; r, host 16S rRNA; ρ, o-16S rRNA; Total, sum of all ribosomes; λ, growth rate. **(A)** Impact of orthogonal ribosomes production on host genes in the absence of circuit genes (i.e. $\omega_G = 0$). **(B)** Impact of orthogonal ribosome production on a circuit gene which utilises the host ribosome pool (i.e. $\omega_G = 100$). Inset in host proteome: Simulations across a o-rRNA induction of 100x ω_ρ. **(C)** Impact of using the orthogonal ribosome pool for circuit gene expression (i.e. $\omega_G = 100$).

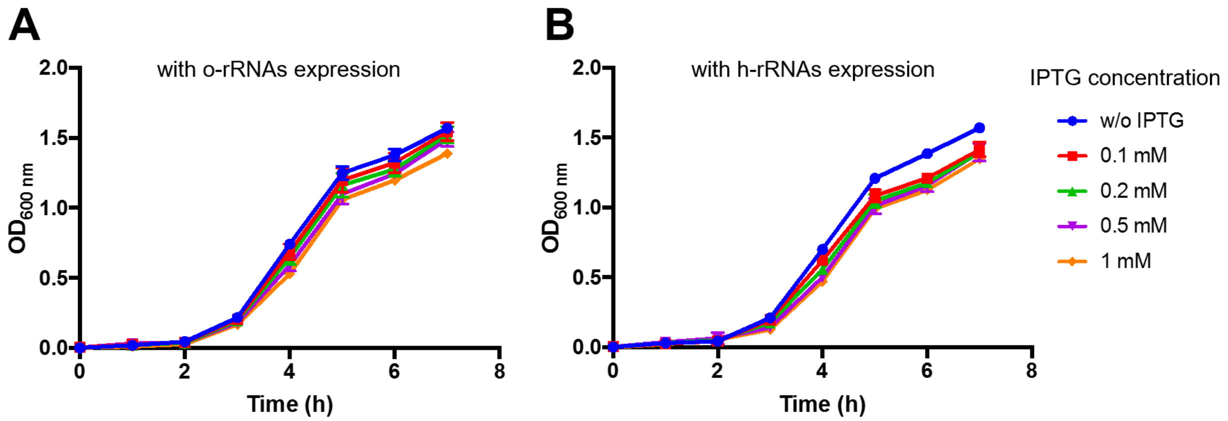

Supplementary Figure 4: **The effect of the accumulation of orthogonal ribosomes on cellular growth.** Growth of the MG1655 strain harbouring orthogonal or host rRNA expression system was monitored over time. Overnight grown cells were diluted into fresh LB medium and grown to the exponential phase. 2 μL of culture was transferred into 1 mL fresh medium containing IPTG at the stated concentration. OD_{600} was recorded using a microplate reader. **(A)** o-16S rRNA expression system. **(B)** host 16S rRNA expression system.

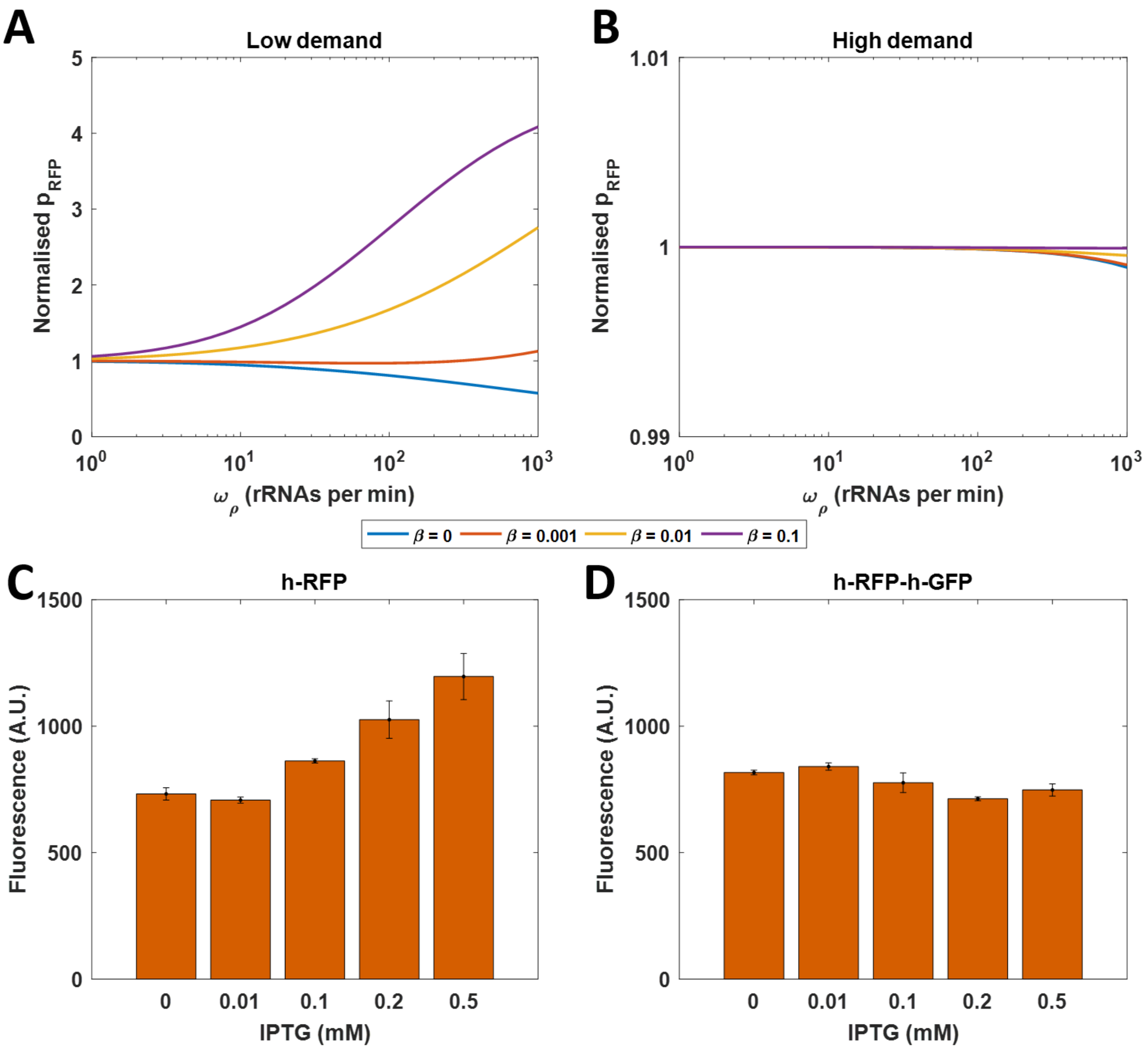

Supplementary Figure 5: **Crosstalk has little effect in circuits with high demand.** Assessment of the impact of crosstalk on circuits with low and high demand. β indicates the level of crosstalk. See Supplementary Note 3 for further discussion. **(A)** Simulations of a low demand circuit. A single gene is expressed utilising the host ribosome pool with low transcription rate $\omega_{RFP} = 10$ mRNAs per min and weak RBS, $b_{RFP} = 0.1$. **(B)** Simulations of a high demand circuit. A single gene is expressed using the host ribosome pool with high transcription rate $\omega_{RFP} = 1000$ mRNAs per min and strong RBS, $b_{RFP} = 1$. **(C)** Experimental low demand circuit. Steady state mean RFP expression using the host ribosome pool, $\pm$ 1 S.D. Data as in Fig. 1B. **(D)** Experimental high demand circuit. Steady state mean h-RFP from the h-RFP-h-GFP circuit. $\pm$ 1 S.D. Data as in Fig. 2B.

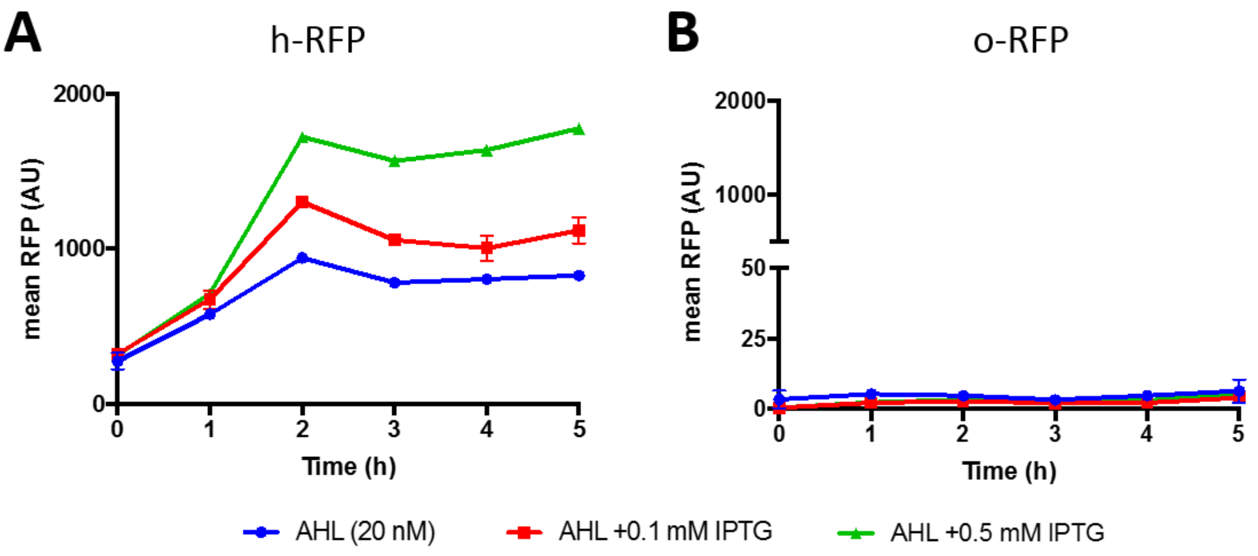

Supplementary Figure 6: **Effect of over expression of the host 16S rRNA on host translated and orthogonally translated genes.** Using the host rRNA expression system, the overexpression of the host rRNA was induced by IPTG at the concentration shown. **(A)** h-RFP. **(B)** o-RFP.

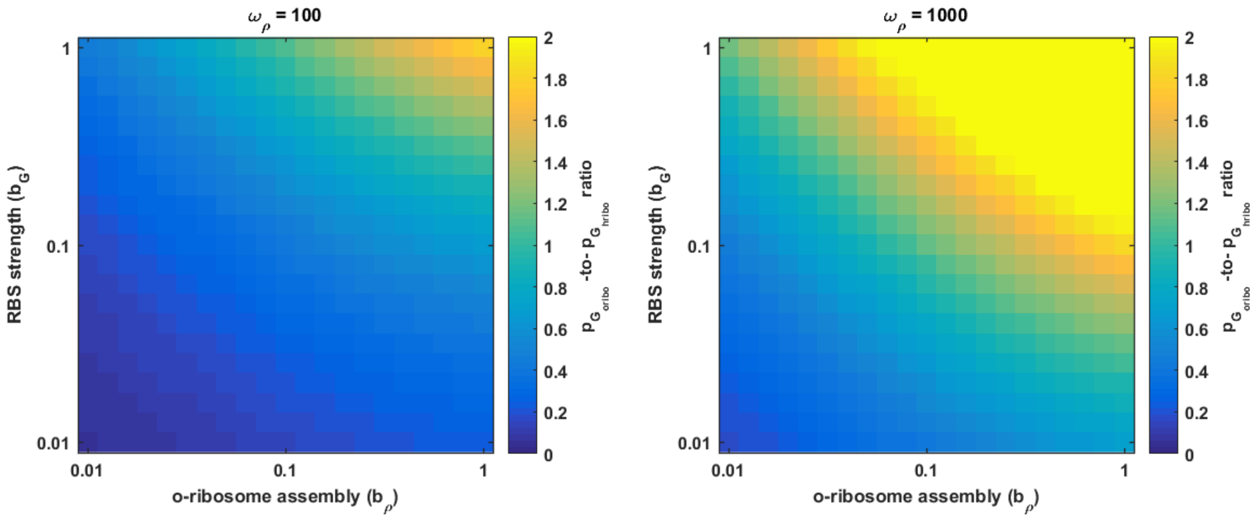

Supplementary Figure 7: **Orthogonal ribosome assembly may be inefficient.** Simulations of the steady state protein outputs for different RBS strengths (b_G-to-u_G ratio) and assembly of the orthogonal ribosome (b_ρ-to-u_ρ ratio) for the given orthogonal ribosome induction (ω_ρ, molecules per min). Circuit gene is induction is constant at $\omega_G = 100$ molecules per min throughout. Results are reported as a ratio of the production utilising the host ribosome pool for the same circuit, with values greater than 2 truncated to 2. See Supplementary Note 4 for further discussion.

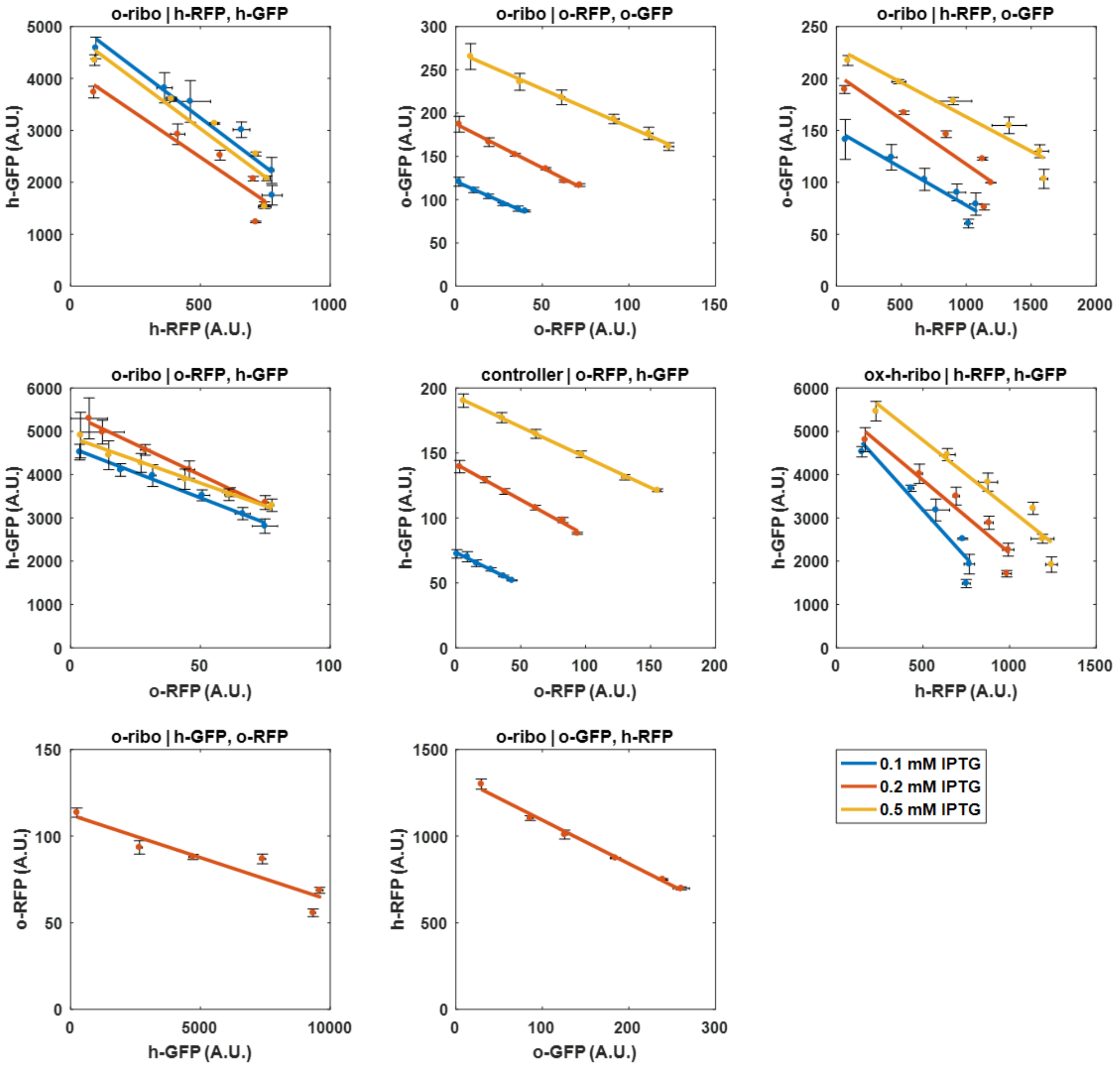

Supplementary Figure 8: **Isocost lines of the gene circuits used in this study.** Isocost lines for each circuit were determined by fitting a straight line through the mean steady state fluorescence. Error bars represent ±1 S.D.. The induced gene output is plotted on the x-axis and the constitutive gene output is plotted on the y-axis. IPTG was varied as shown to change the size of the o-ribosome pool. Note the different scales on each subplot. Plots are titled as follows X | Y, where X represents the strain and Y represents the circuit. The strains are named as follows: oribo, o-ribosome producing strain; controller, o-ribosome producing strain where o-rRNA production is under control of the o-lacI; ox-h-ribo, host 16S rRNA over-expressing strain.

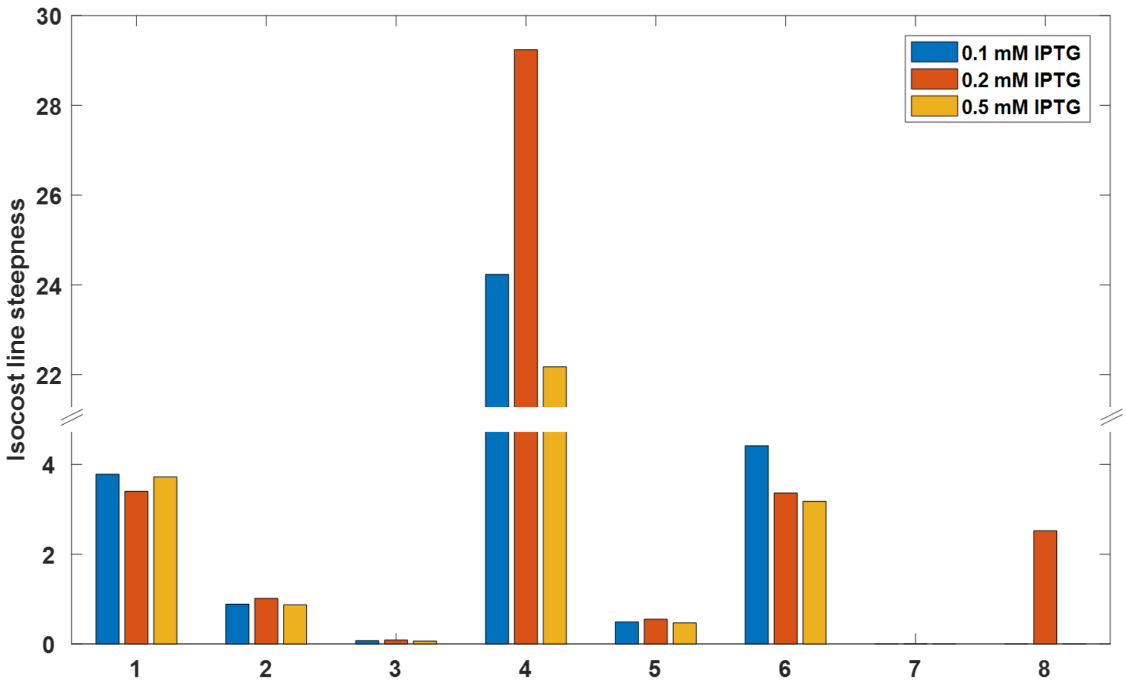

Supplementary Figure 9: **Comparison of experimentally determined isocost lines.** Absolute gradient of the isocost lines for each circuit tested in this study as calculated from data presented in Fig. 8. The bars represent the strains and circuits at each IPTG level shown as follows: 1, o-ribo | h-RFP, h-GFP; 2, o-ribo | o-RFP, o-GFP; 3, o-ribo | h-RFP, o-GFP; 4, o-ribo | o-RFP, h-GFP; 5, controller | o-RFP, o-GFP; 6, ox-h-ribo | h-RFP, h-GFP; 7, o-ribo | h-GFP, o-RFP; 8, o-ribo | o-GFP, h-RFP.

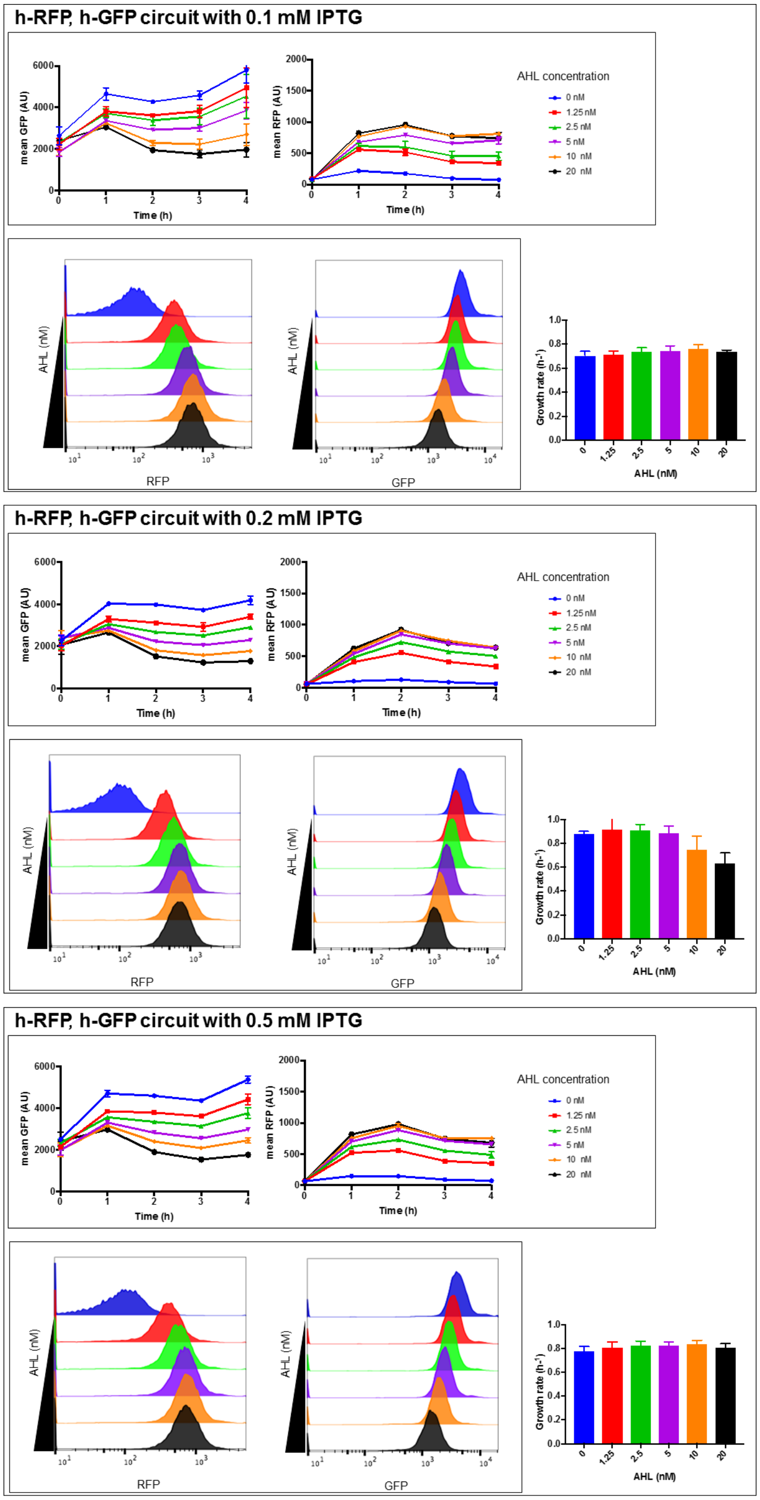

Supplementary Figure 10: **Kinetics of fluorescent reporters production and cell growth of the o-ribosome producing strain carrying the h-RFP, h-GFP circuit**
Kinetics for the circuit at IPTG concentration shown. In each case the time-series analysis of GFP and RFP expression in response to different concentrations of AHL is depicted (upper subpanels). The mean of the fluorescent intensities and the standard deviation from three biological replicates were measured by flow cytometry. Histograms of fluorescent reporter production in the population at the steady state are also shown (lower subpanels). 20,000 cells from each sample were analysed. Bar plots represent growth rates.

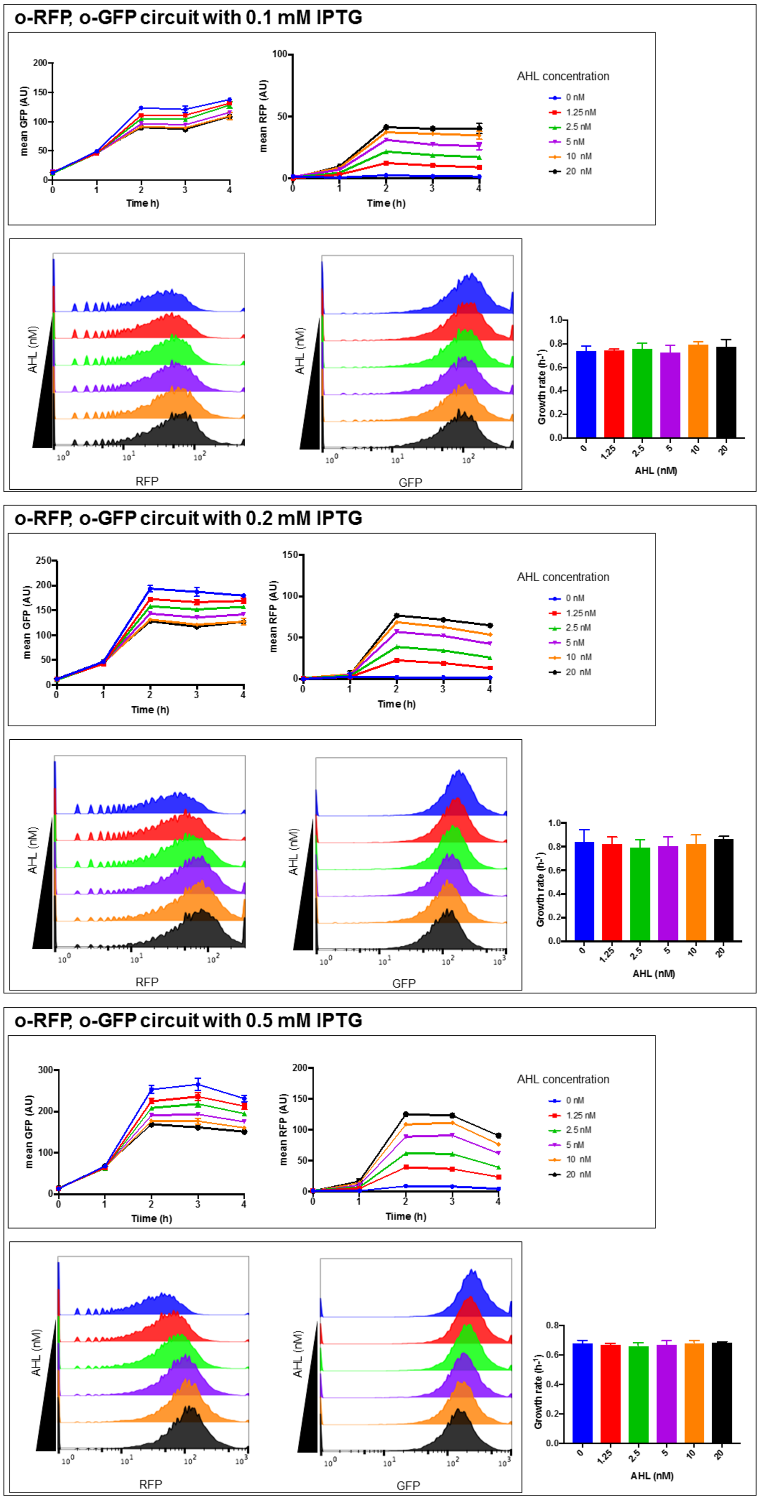

Supplementary Figure 11: **Kinetics of fluorescent reporters production and cell growth of the o-ribosome producing strain carrying the o-RFP, o-GFP circuit**
Kinetics for the circuit at IPTG concentration shown. In each case the time-series analysis of GFP and RFP expression in response to different concentrations of AHL is depicted (upper subpanels). The mean of the fluorescent intensities and the standard deviation from three biological replicates were measured by flow cytometry. Histograms of fluorescent reporter production in the population at the steady state are also shown (lower subpanels). 20,000 cells from each sample were analysed. Bar plots represent growth rates.

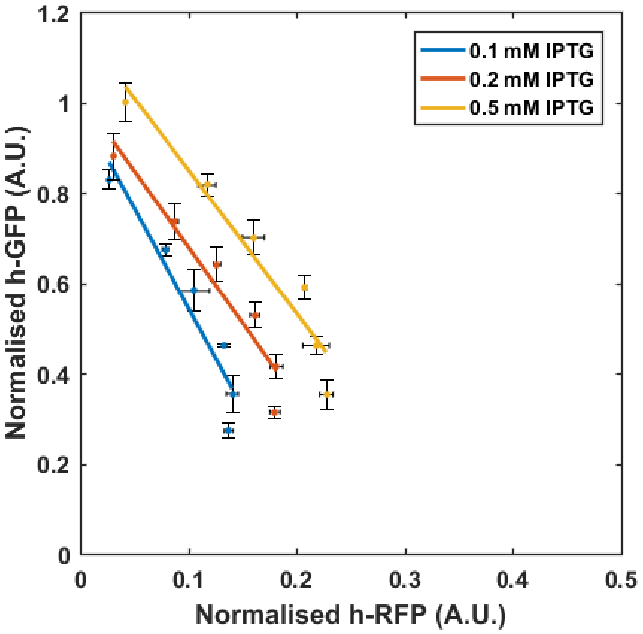

Supplementary Figure 12: **Over expression of host 16S rRNA does not improve coupling.** The wild type host 16S rRNA was overexpressed by induction with IPTG at the levels shown from the host expression system. Coupling is assessed by inducing RFP with AHL from 0 to 20 nM. Points are mean steady state fluorescence ±1 S.D. normalised by maximum GFP expression obtained across different levels of IPTG treatment. $N = 3$. Raw data is shown in Supplementary Fig. 8. Fluorescence was determined by FACS from cultures at 3 hours post induction during mid-exponential growth. The isocost line is fit to the mean fluorescence values and gradients calculated shown in Supplementary Fig. 9. FACS profiles are shown in Supplementary Fig. 13.

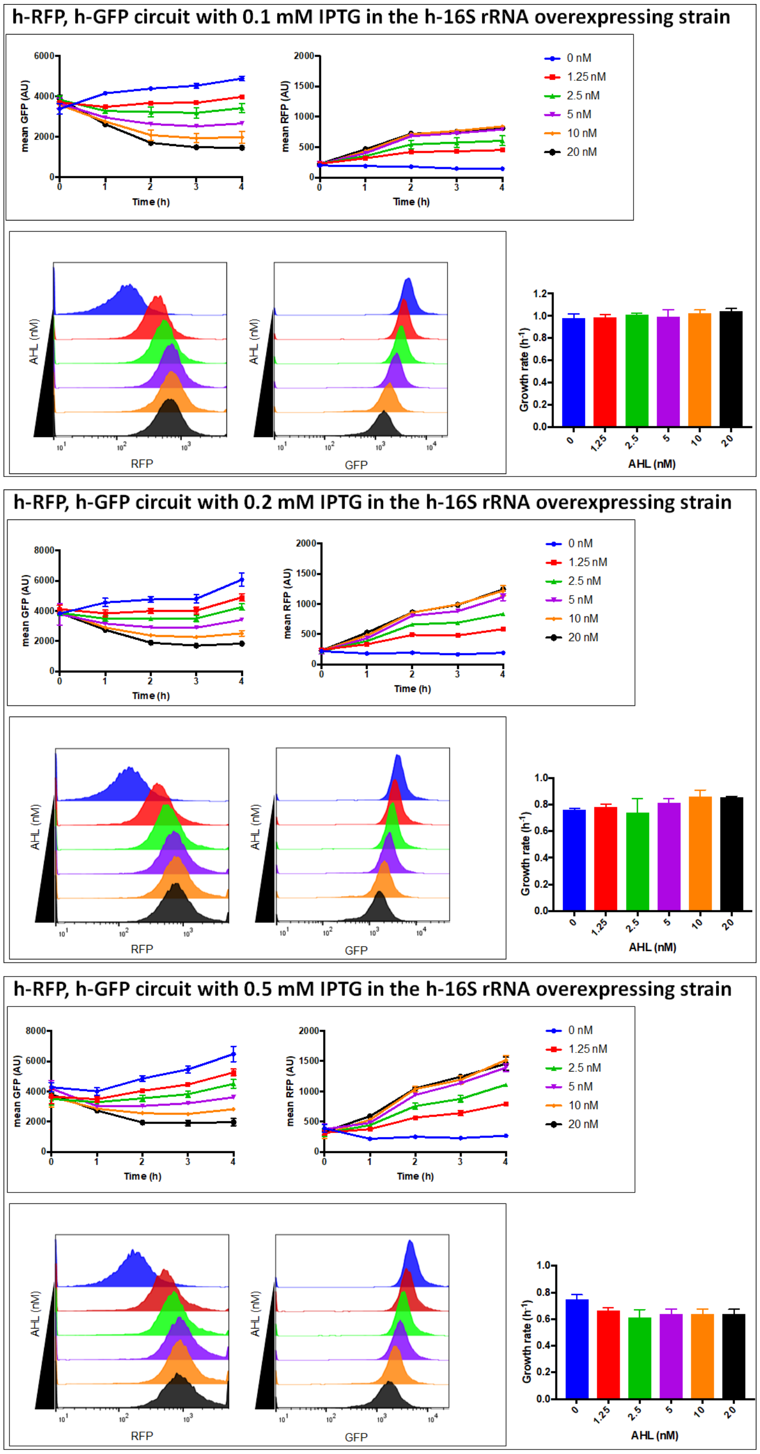

Supplementary Figure 13: **Kinetics of fluorescent-reporters production and cell growth of the host 16S rRNA over-expressing strain carrying the h-RFP, h-GFP circuit**
Kinetics for the circuit at IPTG concentration shown. In each case the time-series analysis of GFP and RFP expression in response to different concentrations of AHL is depicted (upper subpanels). The mean of the fluorescent intensities and the standard deviation from three biological replicates were measured by flow cytometry. Histograms of fluorescent reporter production in the population at the steady state are also shown (lower subpanels). 20,000 cells from each sample were analysed. Bar plots represent growth rates.

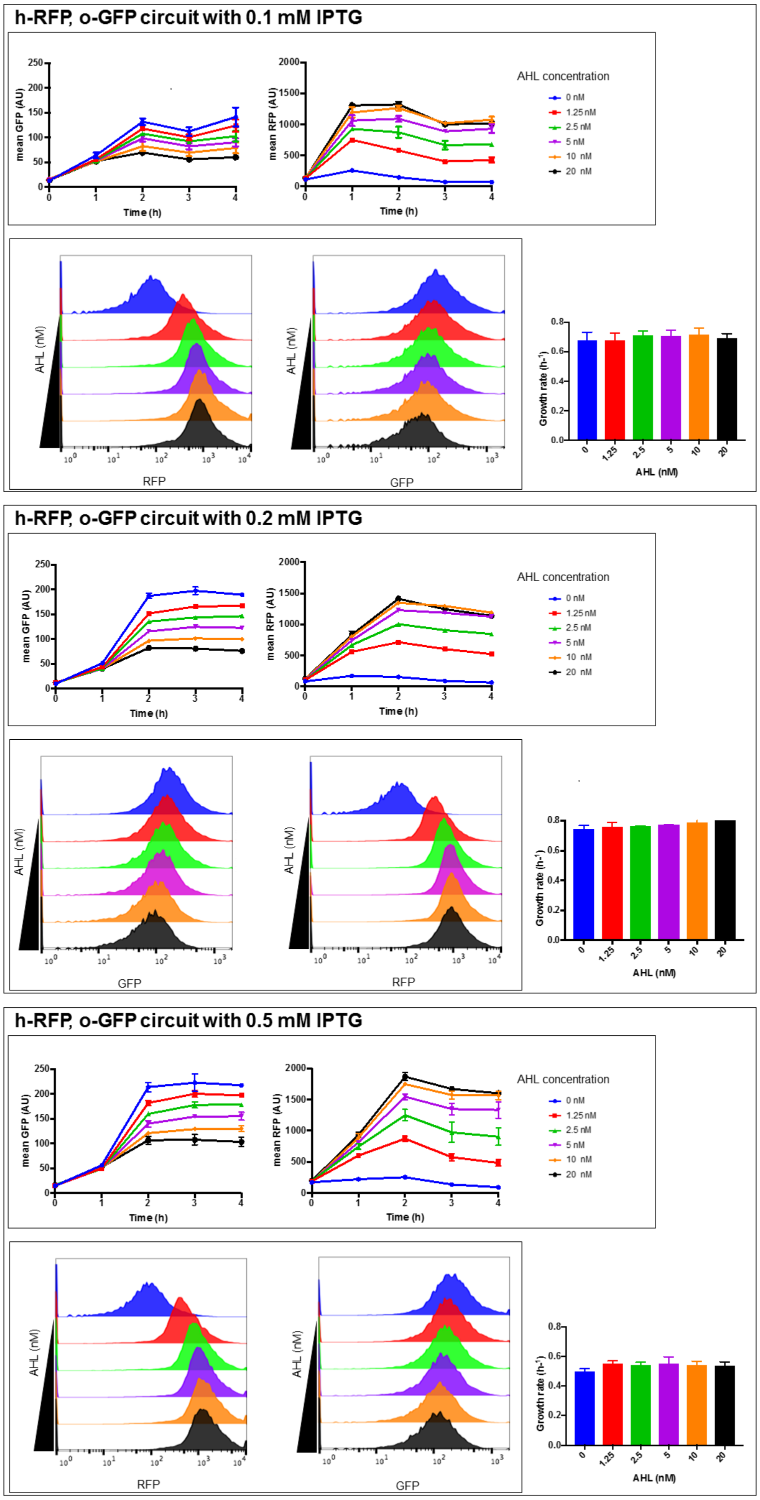

Supplementary Figure 14: **Kinetics of fluorescent reporters production and cell growth of the o-ribosome producing strain carrying the h-RFP, o-GFP circuit**
Kinetics for the circuit at IPTG concentration shown. In each case the time-series analysis of GFP and RFP expression in response to different concentrations of AHL is depicted (upper subpanels). The mean of the fluorescent intensities and the standard deviation from three biological replicates were measured by flow cytometry. Histograms of fluorescent reporter production in the population at the steady state are also shown (lower subpanels). 20,000 cells from each sample were analysed. Bar plots represent growth rates.

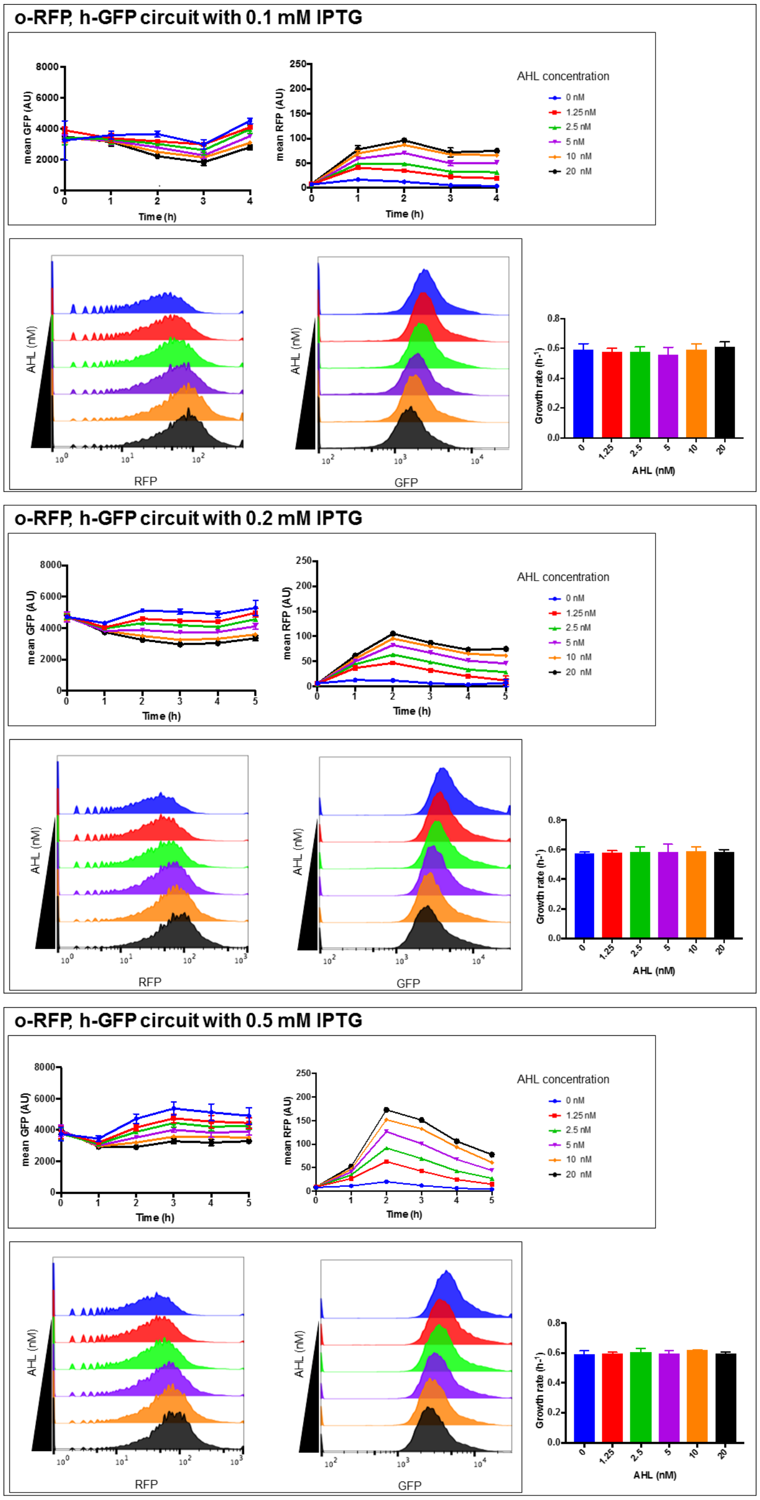

Supplementary Figure 15: **Kinetics of fluorescent reporters production and cell growth of the o-ribosome producing strain carrying the o-RFP, h-GFP circuit**

Kinetics for the circuit at IPTG concentration shown. In each case the time-series analysis of GFP and RFP expression in response to different concentrations of AHL is depicted (upper subpanels). The mean of the fluorescent intensities and the standard deviation from three biological replicates were measured by flow cytometry. Histograms of fluorescent reporter production in the population at the steady state are also shown (lower subpanels). 20,000 cells from each sample were analysed. Bar plots represent growth rates.

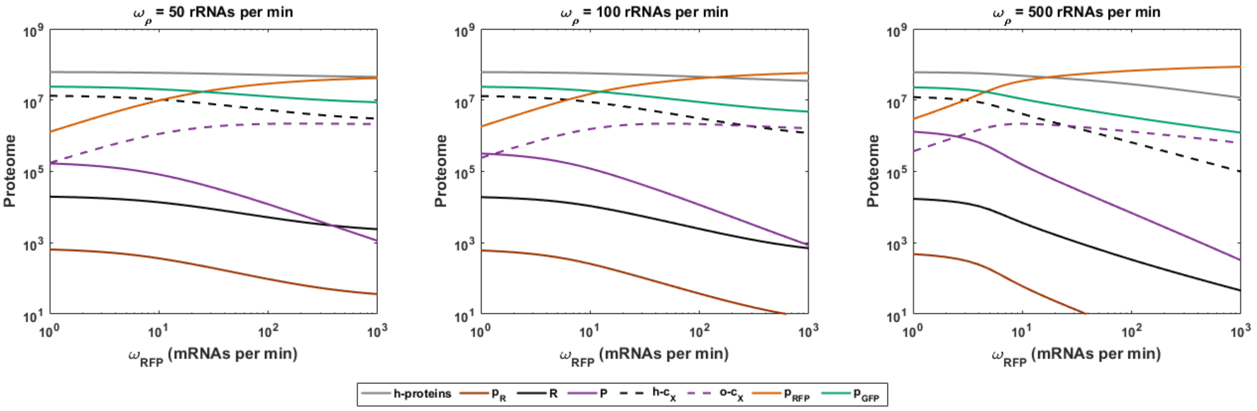

Supplementary Figure 16: **Competition for 'empty ribosomes' explains increased coupling observed in o-RFP, h-GFP circuits.** The distribution of proteome components changes as p_{RFP} increases. The o-rRNA transcription rate is as shown. See Supplementary Note 5 for further discussion. Legend explanation: h-proteins, host proteins; p_R, empty ribosomes; R, host ribosomes; P, orthogonal ribosomes; $h - c_X$, translating host ribosomes; $o - c_X$, translating o-ribosomes; p_{RFP}, RFP protein; p_{GFP}, GFP protein.

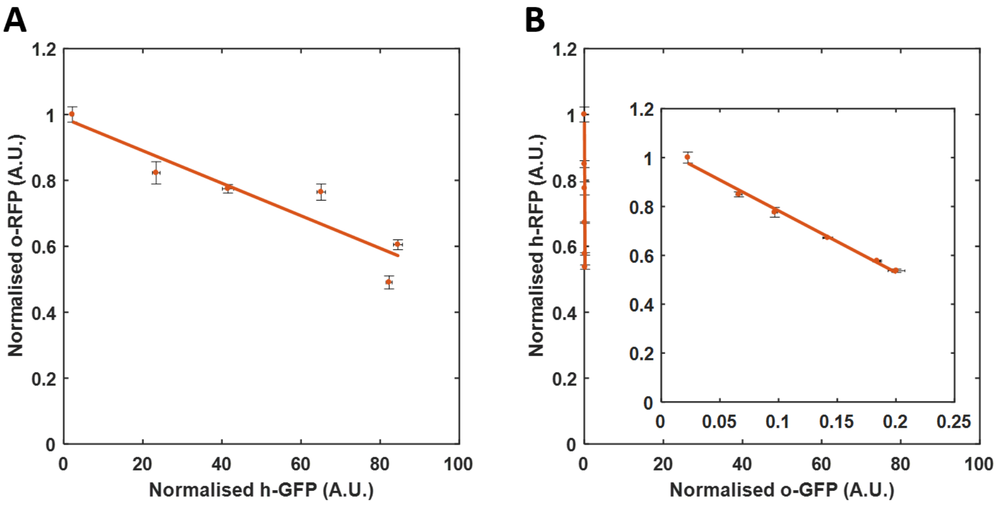

Supplementary Figure 17: **Swapping reporter proteins does not qualitatively change the coupling relationships.** The reporter genes in circuits utilising the host and orthogonal ribosome pools where swapped such that GFP was induced with AHL and RFP was constitutively expressed. GFP was induced using AHL from 0 to 20 nM. Points are mean steady state fluorescence ± 1 S.D. normalised by maximum GFP expression. Raw data is shown in Supplementary Fig. 8. The o-ribosome pool was induced with 0.2 mM IPTG. The isocost line is fit to the mean fluorescence as determined by FACS from cultures at 5 h (Panel A) or 3 h (Panel B) post induction during mid-exponential growth. Gradients calculated are shown in Supplementary Fig. 9. **(A)** h-GFP, o-RFP circuit. FACS profiles are shown in Supplementary Fig. 18. **(B)** o-GFP, h-RFP circuit. The inset shows the data on an expanded x-axis. FACS profiles are shown in Supplementary Fig. 18.

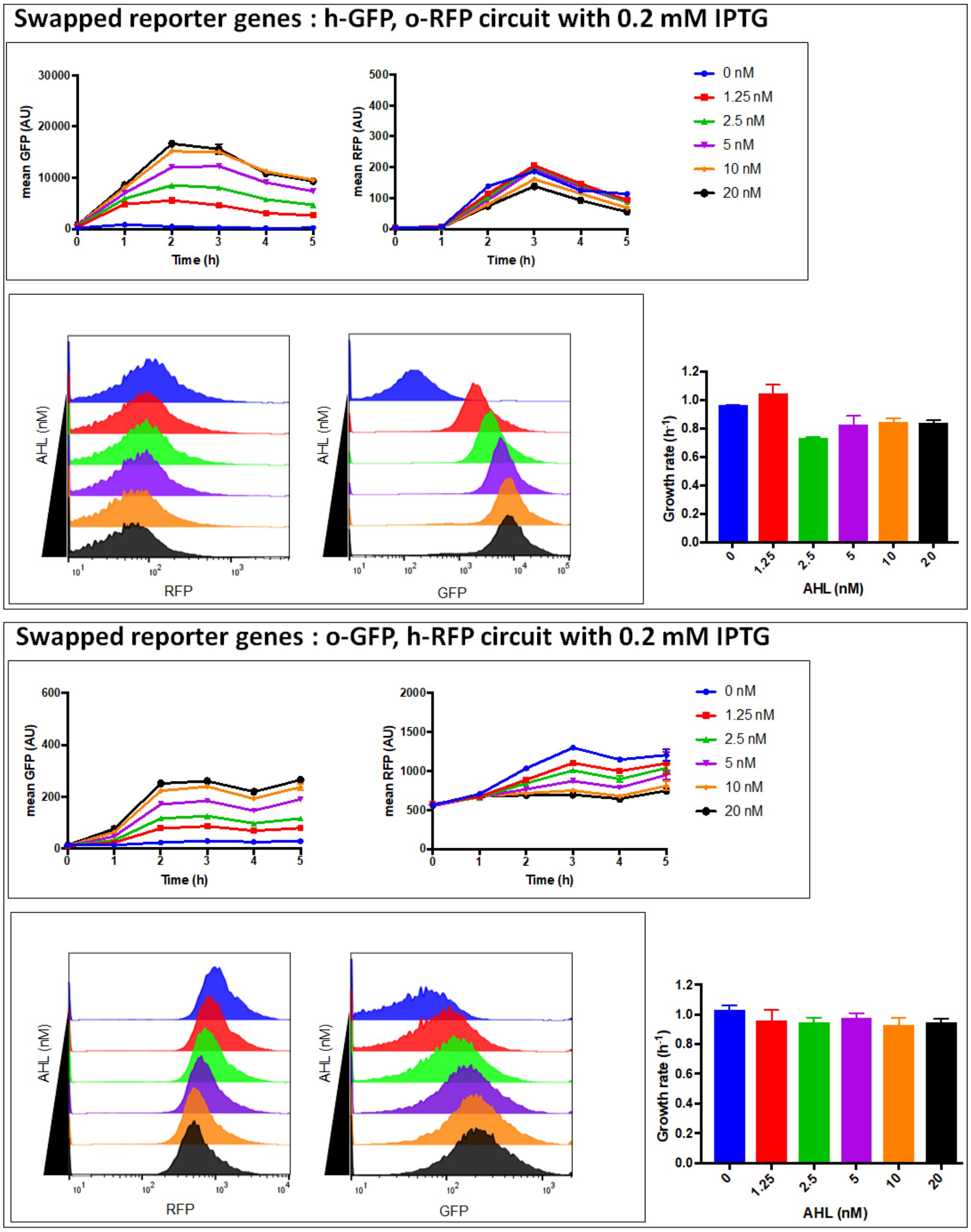

Supplementary Figure 18: **Kinetics of fluorescent-reporters production and cell growth of the o-ribosome producing strain carrying the swapped plasmids h-GFP, o-RFP or o-GFP, h-RFP** Kinetics for the circuit at IPTG concentration shown. In each case the time-series analysis of GFP and RFP expression in response to different concentrations of AHL is depicted (upper subpanels). The mean of the fluorescent intensities and the standard deviation from three biological replicates were measured by flow cytometry. Histograms of fluorescent reporter production in the population at the steady state are also shown (lower subpanels). 20,000 cells from each sample were analysed. Bar plots represent growth rates.

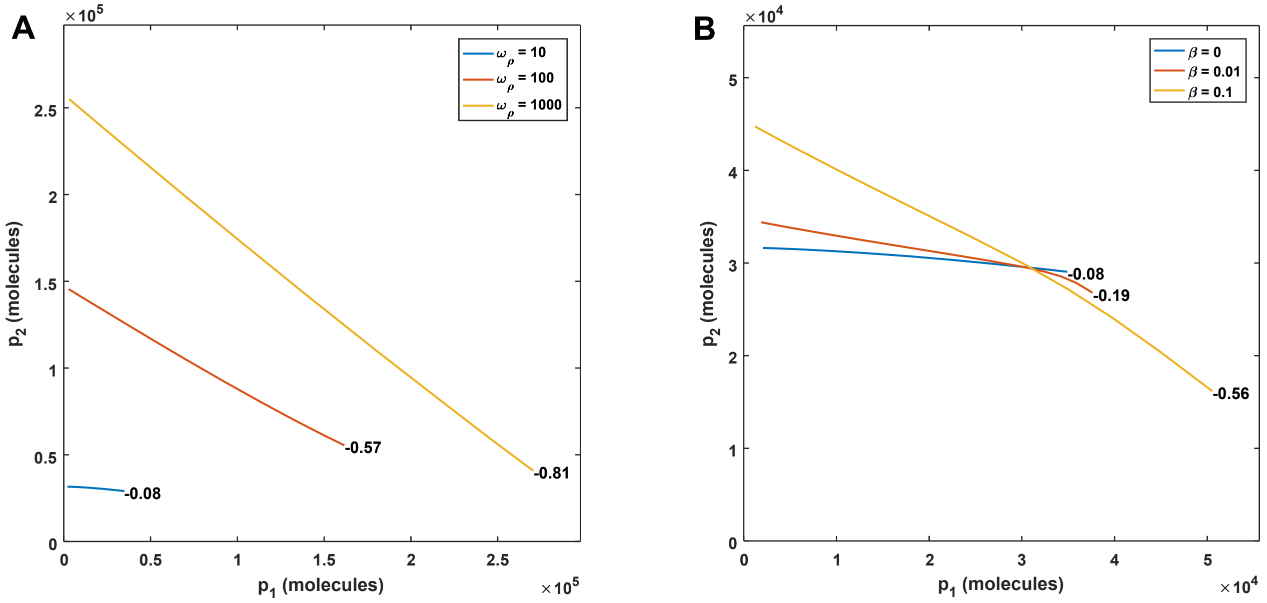

Supplementary Figure 19: **Gene coupling in gene circuits utilising multiple orthogonal ribosome pools.** Simulations of the steady state protein outputs of a simple two gene circuit where each gene utilises its own orthogonal ribosome pool. See Supplementary Note 6 for further discussion. **(A)** Isocost lines at different orthogonal ribosome production rates (ω_ρ). Numbers represent the slope of the isocost line. **(B)** Simulations of the optimal o-rRNA production rates ($\omega_\rho = 10$ molecules per min) with the introduction of cross talk (β).

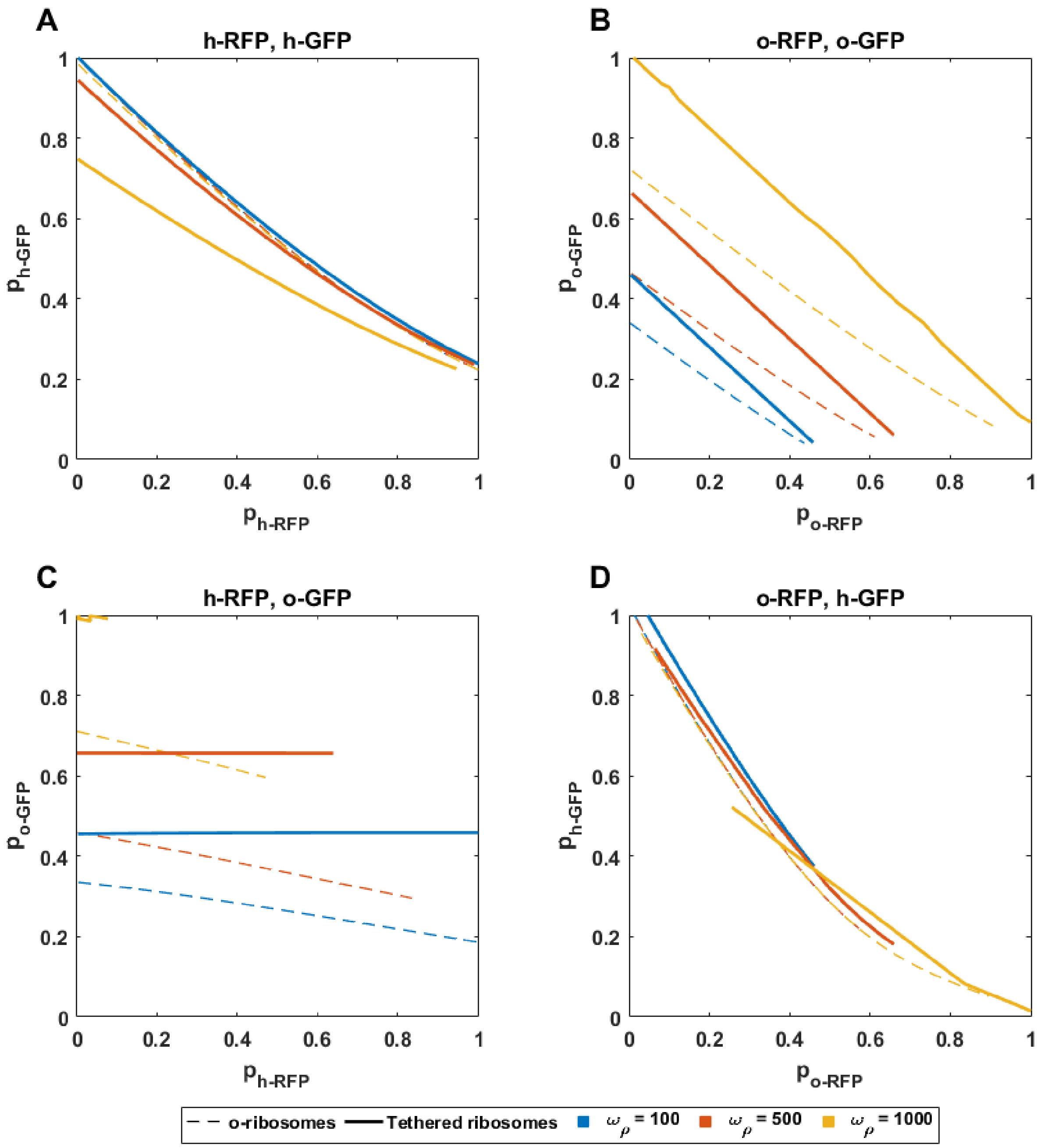

Supplementary Figure 20: **Tethered o-ribosomes may increase yields and increase decoupling in some contexts.** Simulations of the steady state concentrations of RFP and GFP normalised by the maximum protein production achieved across the o-rRNA transcription rates tested where translation is carried out by tethered orthogonal ribosomes (solid line, $b_\rho = 0.45$ and $u_\rho = 0$). For comparison simulations of non-tethered orthogonal ribosomes are also shown (dashed lines, $b_\rho = u_\rho = 1$). $\omega_{GFP} = 100$ and $\omega_{RFP} = 1$ to 10^3 mRNAs per min. o-rRNA production (ω_ρ) was simulated at the RNAs per min as shown. See Supplementary Note 7 for further discussion.

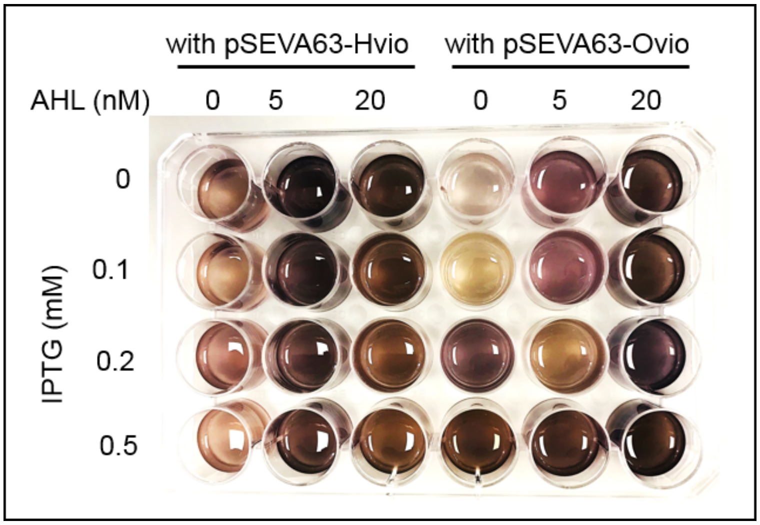

Supplementary Figure 21: **Representative plate showing violacein production at 24 h post induction with AHL.** Cells were grown in LB supplemented with tryptophan as outlined in the methods. The downstream *vioBCDE* cassette and orthogonal ribosome pool was induced with AHL and IPTG respectively at the concentration shown.

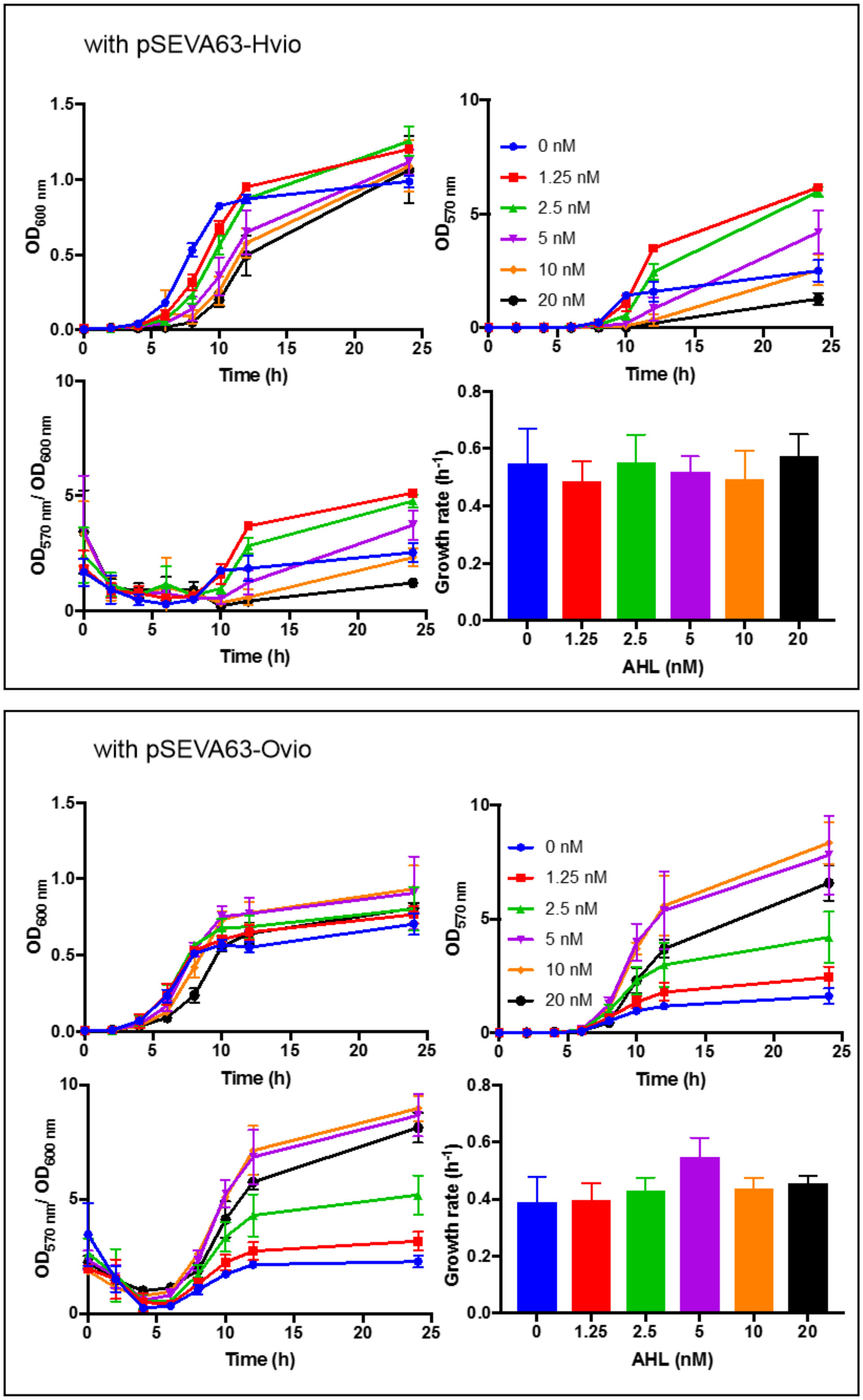

Supplementary Figure 22: **Time series analysis of violacein production and cell growth of the strain carrying either the Hvio or Ovio circuit.** Violacein production over time in response to different concentrations of AHL was determined spectrophotometrically at 570 nm after extraction. The growth profile was obtained by measuring cell debris at OD_600 nm after extraction of the pigment as described in the Methods.

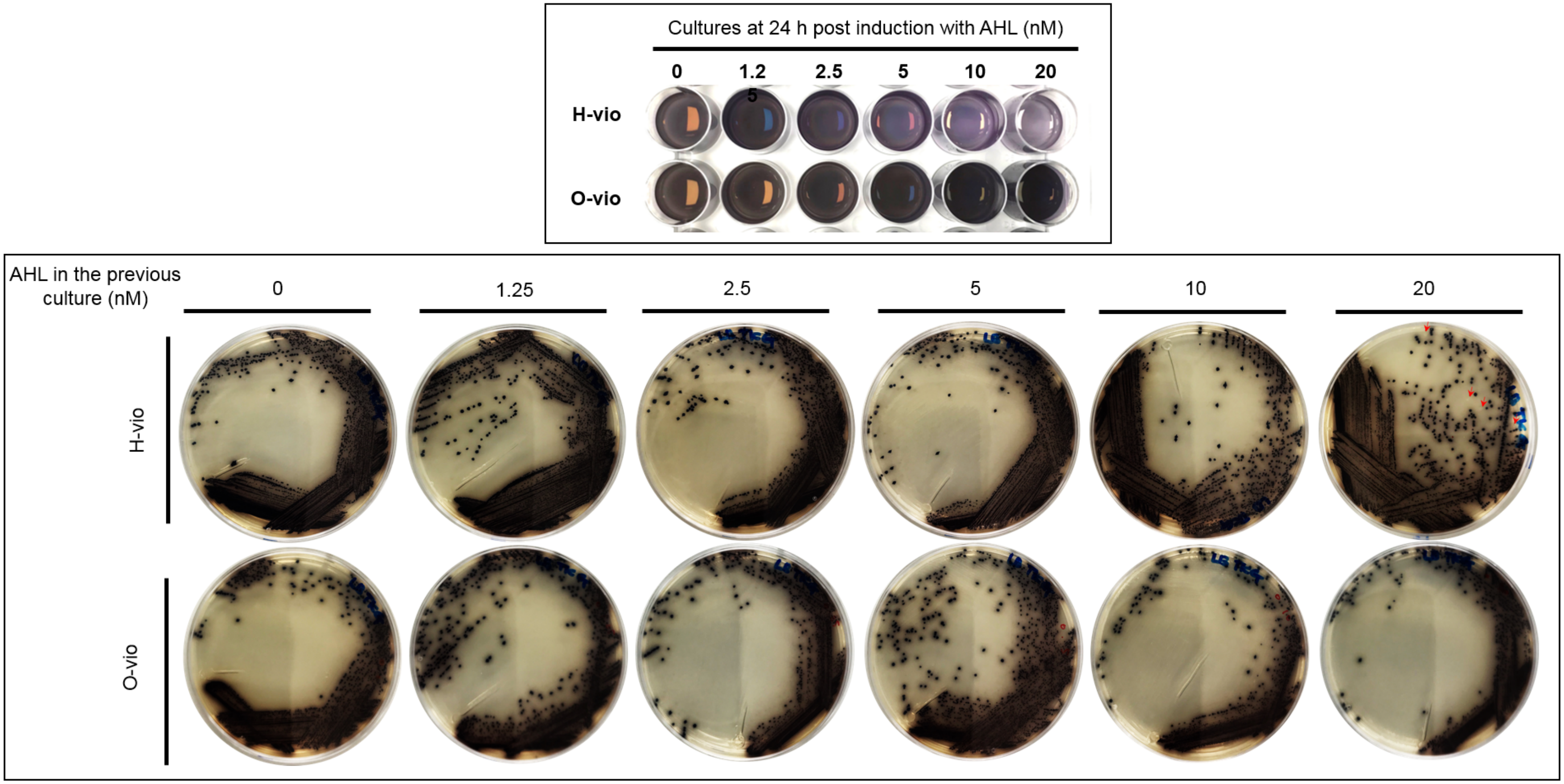

Supplementary Figure 23: **The emergence of mutants in the violacein pathway is negligible after 24 h growth.** Violacein producing cultures were grown in the presence of the AHL concentration shown. o-ribosome production was induced with 0.2 mM IPTG. The final cultures after 24 h are shown *(upper panel)*. Cells recovered from each culture condition and streaked on LB supplemented with tryptophan 1 g/L (and no AHL) to assess the presence of mutants which have lost the ability to produce violacein (i.e. screening for white colonies) *(lower panel)*. Only four white colonies (denoted with red arrows), which correspond to the highest AHL concentration tested for the H-vio circuit, were identified in this screening.

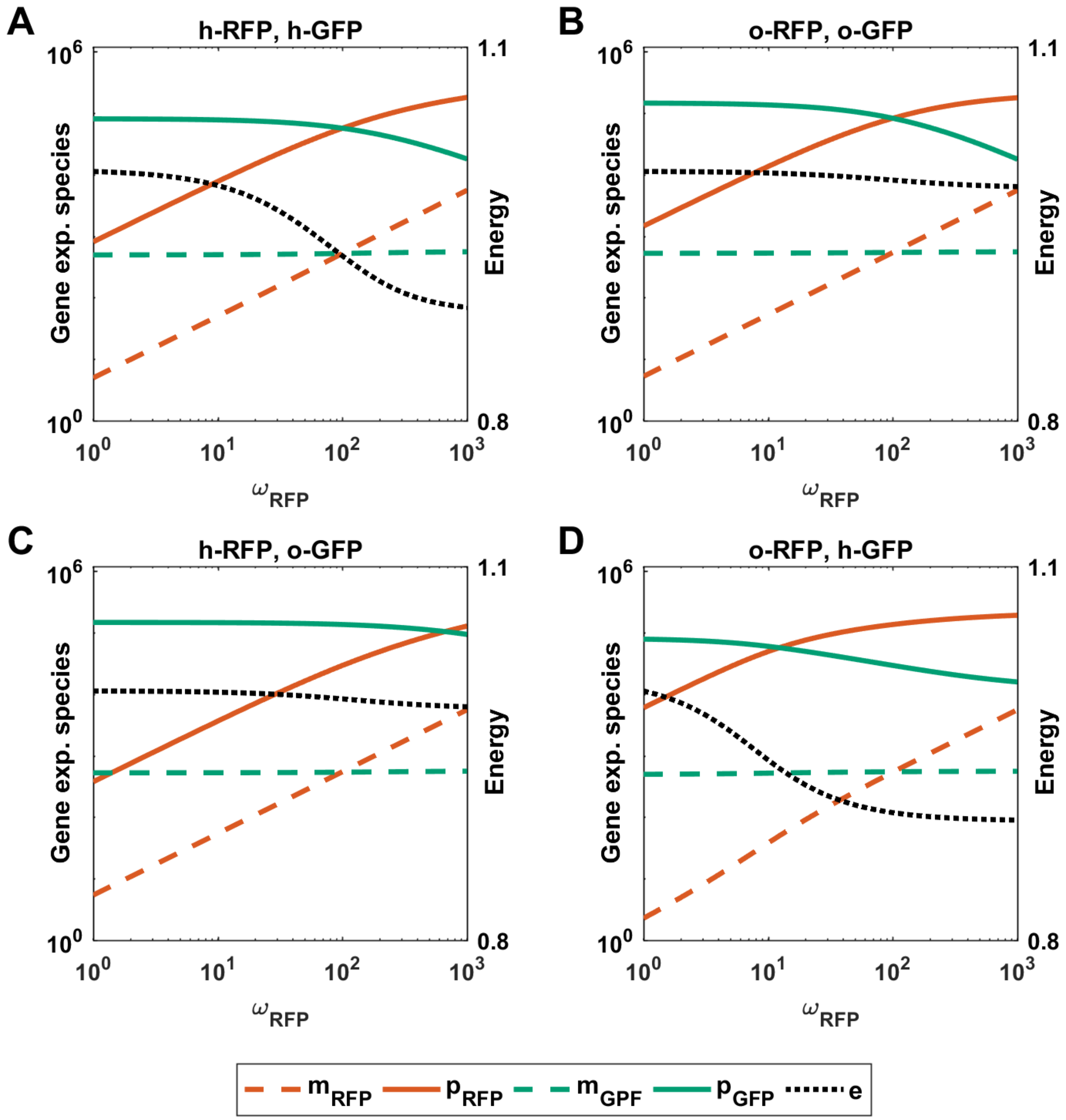

Supplementary Figure 24: **Gene expression plateauing is mediated by saturation of the ribosome pool.** Simulations of the steady state concentrations of RFP and GFP mRNAs and protein levels. See Supplementary Note 8 for further discussion. $\omega_{GFP} = 100$ and $\omega_{RFP} = 1$ to 10^3 mRNAs per min. Only one o-rRNA transcription rate is shown ($\omega_\rho = 100$ rRNAs per min). Normalised levels of the intermediate 'energy' metabolite (e) are also shown. **(A)** Both genes utilising the host ribosome pool. **(B)** Both genes utilising the o-ribosome pool. **(C)** Induced RFP translated by the host pool whilst the constitutively expressed GFP is translated by the o-ribosome pool. **(D)**. Induced RFP translated by the o-ribosome pool whilst the consitutively expressed GFP is translated by the host ribosome pool.

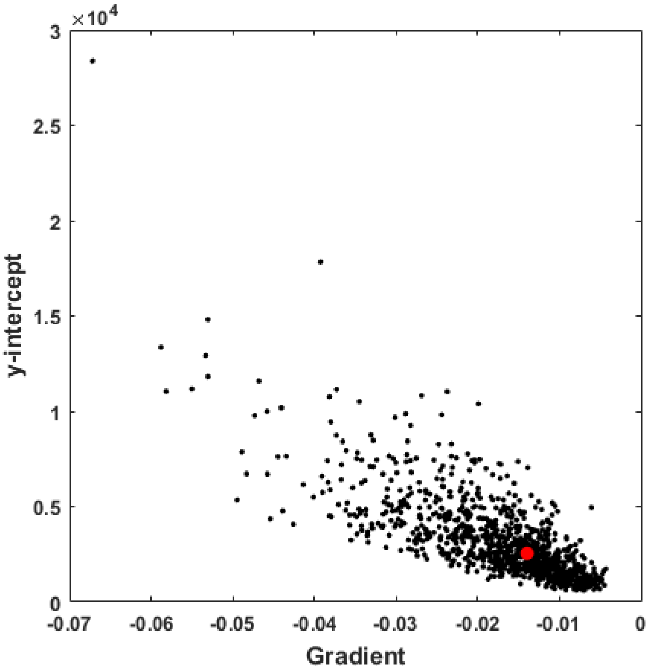

Supplementary Figure 25: **Trade-off in controller design.** Isocost lines were fit to the result of each simulation in the robustness analysis. The gradient and y-intercept are shown. The optimal parameter set is highlighted in red.

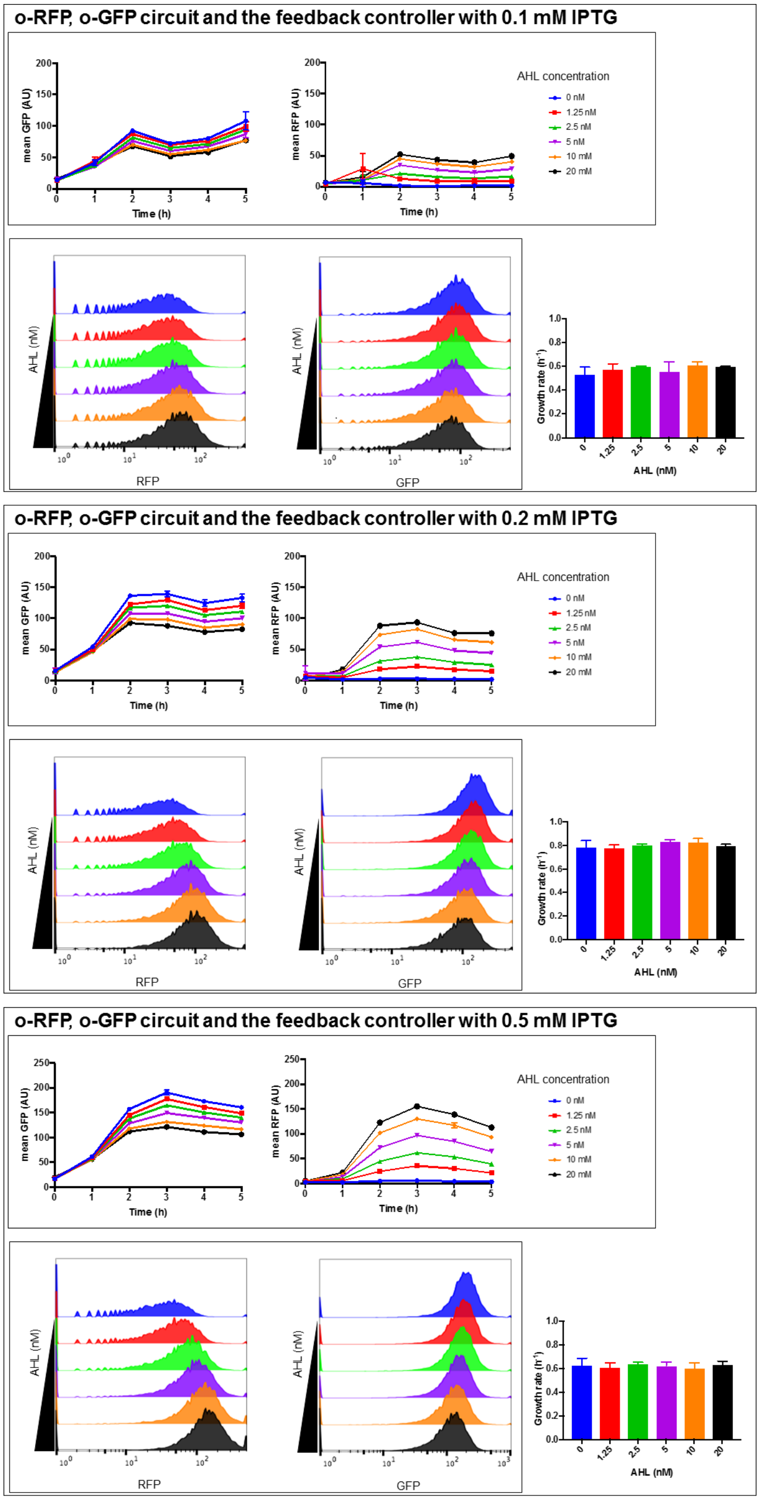

Supplementary Figure 26: **Kinetics of fluorescent reporters production and cell growth of the o-ribosome producing strain carrying the o-RFP, o-GFP circuit and feedback controller**
Kinetics for the circuit at IPTG concentration shown. In each case the time-series analysis of GFP and RFP expression in response to different concentrations of AHL is depicted (upper subpanels). The mean of the fluorescent intensities and the standard deviation from three biological replicates were measured by flow cytometry. Histograms of fluorescent reporter production in the population at the steady state are also shown (lower subpanels). 20,000 cells from each sample were analysed. Bar plots represent growth rates.

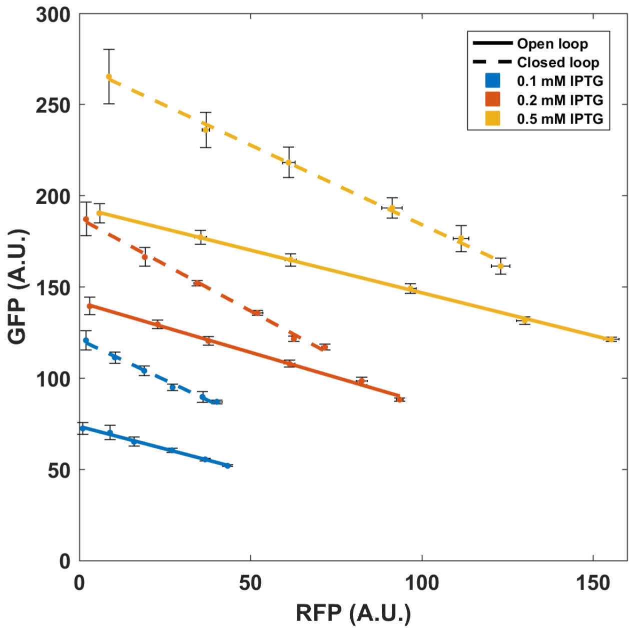

Supplementary Figure 27: **Comparison of open loop and closed loop conformations.** Extended version of Figure 7 showing all IPTG confirmations for both un-controlled (open loop) and controlled system (closed loop). This data is the same as in Supplementary Fig. 8. Response of constitutively expressed o-GFP as o-RFP is induced. o-RFP was induced using AHL from 0 to 20 nM. Points are the mean steady state fluorescence ± 1 S.D. as determined by FACS from cultures during mid-exponential growth (between 3-5 hours post-induction dependent on the strain and circuit). $N = 3$. Isocost lines are fit to mean values as described in the Methods. Note that the same IPTG concentrations do not produce the same initial GFP level (y-intercept).

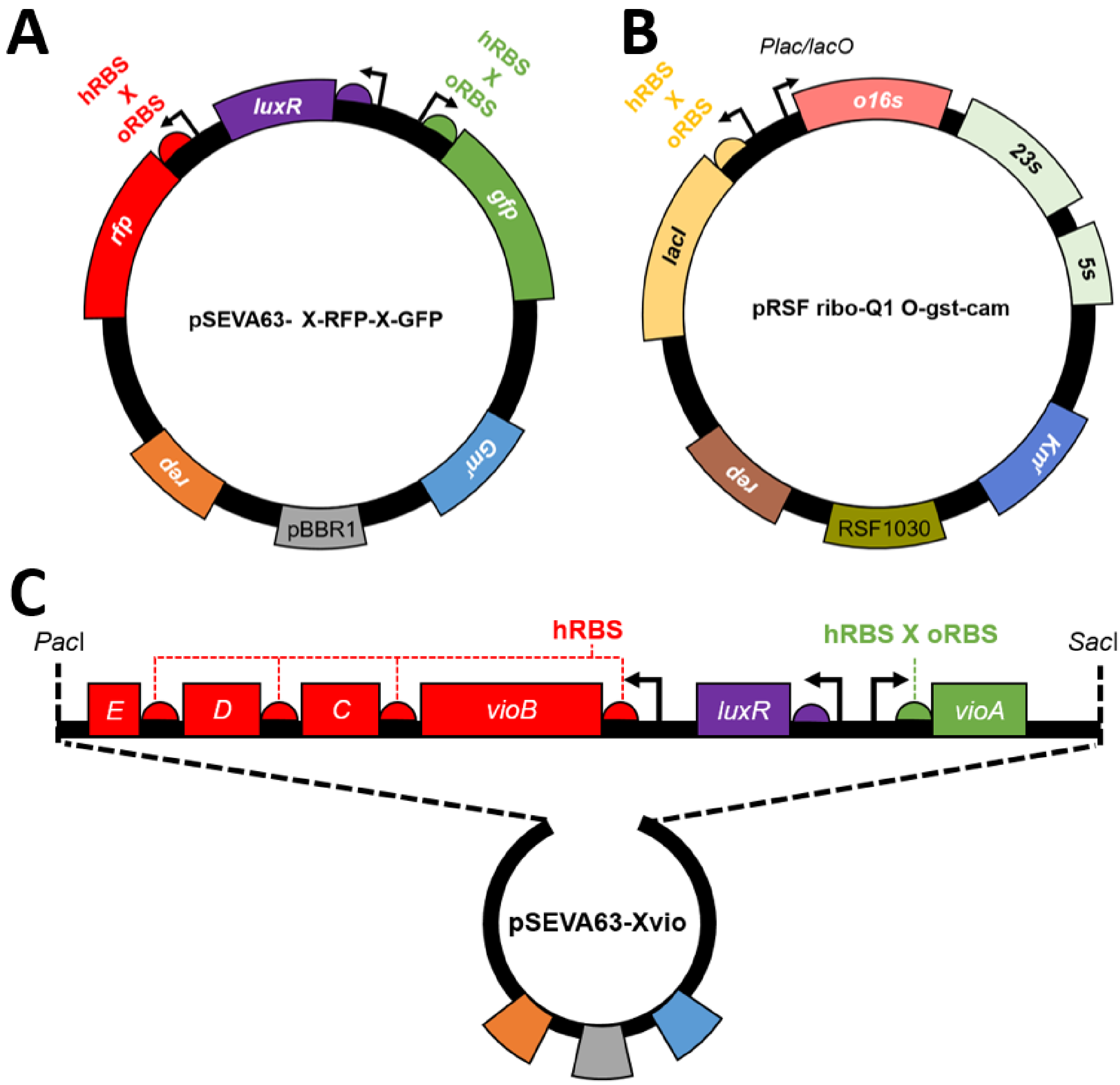

Supplementary Figure 28: **Schematic illustration of the plasmids used in this study.** X means translation by either the host (h-) or orthogonal (o-) ribosome pool. **(A)** Translation of each of the reporter genes *gfp* and *rfp* may be under the control of a canonical ribosome binding site (RBS) or the orthogonal RBS giving rise to four different combinations in which the same plasmid backbone is maintained. **(B)** The expression of rRNAs, which include the orthogonal 16S rRNA, is controlled by the repressor LacI present in the plasmid [3]. The feedback controller is constructed replacing the original RBS of LacI with the orthogonal RBS. **(C)** Plasmid carrying genes which enable violacein production.

***E. coli* strains**	**Description**	**Ref.**
MG1655	F^-, λ^-, *ilvG*$^-$, *rfb-50*, *rph-1*	[4]
MG1655ΔlacIZYA	MG1655 derivate with a full deletion of lacIZYA genes	This study

DH5α	Cloning host: F^- Φ80 lacZΔMΔ15 (*lacZYA-argF*), U169, *recA1*, *endA1*, *hsdR17*, R^-M^+, *supE44*, *thil*, *gyrA*, *relA1*	[5]
DH5α λpir	DH5α λpir phage lysogen	[6]

**Plasmids**	**Description**	**Ref.**
pRSF ribo-Q1 o-gst-cam	Vector carrying an orthogonal 16S rRNA and host 23S and 5S rRNA under the P_{lac} promoter regulated by LacI.	[3]
pRSF ribo-Q1 o-gst-cam o-lacI	pRSF ribo-Q1 o-gst-cam derivative containing an orthogonal RBS for the lacI gene	This study
pEMG	Suicide plasmid, Km^R, oriR6K, *lacZ*α with two flanking I-SceI sites	[6]
MBP 1.0	Vector carrying a circuit formed by consitutive GPF and inducing RFP reporters.	[7]
pEMG-Dual	Same as pEMG but carrying the circuit present in MBP 1.0	Kim *et al.* , unpublis-hed
pEMG-o-RFP-h-GFP	Modification of pEMG-Dual to include an orthogonal RBS for RFP	This study
pEMG-h-RFP-o-GFP	Modification of pEMG-Dual containing an orthogonal RBS for GFP	This study
pEMG-o-RFP-o-GFP	Modification of pEMG-Dual containing orthogonal RBS for both RFP and GFP	This study
pSEVA631	Gm^r, ori-pBBR1	[8]
pSEVA63-Dual	pSEVA631 carrying the circuit MBP 1.0	This study
pSEVA63-o-RFP-h-GFP	Modification of pSEVA63-Dual containing an orthogonal RBS for RFP	This study
pSEVA63-h-RFP-o-GFP	Modification of pSEVA63-Dual containing an orthogonal RBS for GFP	This study
pSEVA63-o-RFP-o-GFP	Modification of pSEVA63-Dual containing orthogonal RBSs for both RFP and GFP	This study
pSEVA63-h-RFP	Modification of pSEVA63-Dual carrying only the RFP re-porter	This study

pSEVA63-o-RFP	Modification of pSEVA63-h-RFP containing an orthogonal RBS for RFP	This study
pKD4	Template for Km cassette, oriR6Kgamma, *bla*, *aphA*	[9]
pKD46	Red recombinase expression vector, repA101ts, oriR101, P_{araB} *exo*, *bet*, *gam araC bla*	[9]
pCP20	Flp recombinase expression plasmid	[9]
pSEVA63sw-o-GFP-h-RFP	Modification of pSEVA63-o-RFP-h-GFP that the GFP and the RFP gene were swapped	This study
pSEVA63sw-h-GFP-o-RFP	Modification of pSEVA63-h-RFP-o-GFP that the GFP and the RFP gene were swapped	This study
pSEVA63-Hvio	Vector carrying a set of genes enabling production of violacein.The circuit is composed with the *vioA* gene, constitutively expressed, and the *vioBCDE* genes, which are inducible	This study
pSEVA63-Ovio	Modification of pSEVA63-Hvio containing an orthogonal RBS for the *vioA* gene	This study
pRSF ribo-X wt-gst-cam	Vector carrying host 16S, 23S and 5S rRNA under the P_{lac} promoter regulated by LacI	[10]

Supplementary Table 3: **Bacterial strains and plasmids**

**Oligonucleotides**	**Sequence (5'→3')**
Dual F	CGCTTAATTAATAGGCGTATCACGAGGCAGA
Dual R	CGCGAGCTCAACGCGAAGTAATCTTTTCGG
RFP ORBS F	ACAATTTTCATATCCCTCCGCAAATGGCTTCCTCCGAAGACGTTATC
RFP ORBS R	TTGCGGAGGGATATGAAAATTGTGTATTTATTCGACTATAACAAACC
GFP ORBS F	ACAATTTTCATATCCCTCCGCAAATGCGTAAAGGAGAAGAACTTTTC
GFP ORBS R	TTGCGGAGGGATATGAAAATTGTGTAGTGCTCAGTATCTCTATCACT
Dual rfp R	CGCGAGCTCTAGTATGGTGCAAAACCTTTC
pAS2 olacI pt1F	GTATGGCATGATAGCGCCCGGAAGACAATTTTCATATCCCTCCGCAAATGAAACCAGTAACGTTATACGATGTC
pAS2 olacI pt1R	TCAATTTTCAGCTTGATCCAGATTT
pAS2 olacI pt2F	ATCTGGATCAAGCTGAAAATTGA
pAS2 olacI pt2R	CGACTGAGCCTTTCGTTTTATTTGA
pAS2 olacI pt 3F	AAATAAAACGAAAGGCTCAGTCG
pAS2 olacI pt3R	ATGCTCGATGAGTTTTTCTAAG
pAS2 olacI pt4 F	TTCTTAGAAAAACTCATCGAGCAT
pAS2 olacI pt4R	AAATTGTCTTCCGGGCGCTATCATGCCATAC
Lac KO F	GTATGGCATGATAGCGCCCGGAAGAGAGTCAATTCAGGGTGGTGAATGTGGTGTAGGCTGGAGCTGCTTC
Lac KO R	AGCGCAGCGTATCAGGCAATTTTTATAATTTAAACTGACGATTCAACTTTATGGGAATTAGCCATGGTCC
63sw F	GATAGTGCTAGTGTAGATCGCTACTAGTAGCGGCCGCTGCAGTCC
63sw R	ATGAACTATACAAATAATAATACTAGAGTACTAGCTCTAGATGCG
GFPsw F	TAGAGCTAGTACTCTAGTATTATTATTTGTATAGTTCATCCATGC
oGFPsw R	TACACAATTTTCATATCCCTCCGCAAATGCGTAAAGGAGAAGAACTTTTC
oGluxRsw F	TTGCGGAGGGATATGAAAATTGTGTATTTATTCGACTATAACAAACCATT
luxRsw R	TCTTCGGAGGAAGCCATCTAGTATTTCTCCTCTTTCTCTAGTAGT
RFPsw F	TACTAGAGAAAGAGGAGAAATACTAGATGGCTTCCTCCGAAGACGTT
RFPsw R	CTGCAGCGGCCGCTACTAGTAGCGATCTACACTAGCACTATCAGCG
GFPsw R	AATACTAGAGAAAGAGGAGAAATACTAGATGCGTAAAGGAGAAGA
LuXRsw F	CTAGTATTTCTCCTCTTTCTCTAGTATTTATTCGACTATAACAAACCA
oRluxRsw R	TTGCGGAGGGATATGAAAATTGTGTAGTGCTCAGTATCTCTATCACT
oRFPsw F	TACACAATTTTCATATCCCTCCGCAAATGGCTTCCTCCGAAGACGTTATC
ovioA F	TACACAATTTTCATATCCCTCCGCAAATGAAGCATTCTTCCGATATCT
ovioA R	CGCGAGCTCAACGCGAAGTAATCTTTTCGGT
lux-vioA F	TCCGAAGCCATTGTTAGCCGTATG
lux-vioA R	TTGCGGAGGGATATGAAAATTGTGTAGTGCTCAGTATCTCTATCACT

Supplementary Table 4: **Primers**

Restriction enzyme sites and engineered orthogonal ribosome binding site are underlined.

Supplementary Note 1

Model description and numerical methods

Given that our work focuses on influencing the host ribosome pool, we initially developed a simple model of microbial physiology which allows us to assess the effect of orthogonal ribosome production and usage on host physiology. We based our model on the ordinary differential equation model of microbial growth and gene expression trade-offs recently developed by Weiße *et al.* [2]. This base model captures the three fundamental trade-offs in bacterial gene expression, i.e. (i) energy production is limited by substrate import and enzymatic activity, (ii) ribosomes are autocatalytic and compete for other genes for their own expression resulting in a finite translational capacity, and (iii) the proteome mass is finite.

We refine this base model by extending the ribosome biosynthesis reactions to include the production of both protein and ribosomal RNA components. We introduce the necessary species to describe the production of orthogonal rRNAs and partition the ribosome pool. This model allows us to characterise the impact of dividing the cell's translational capacity between host and circuit genes.

Model description

The model represents a simplified microbe with:

1. A minimal metabolism which converts an external substrate (s_e) into energy (e), via an internal substrate (s_i)
2. A minimal transcriptome containing mRNAs (denoted m_X, where X is the gene being encoded) and ribosomal rRNAs (r)
3. A minimal proteome containing transport proteins (p_T), metabolic enzymes (p_E), host proteins (p_H), ribosomal proteins (p_R) and functional host ribosomes (R)
4. A simplified ribosome biosynthesis scheme which describes the formation of ribosomes (R) from host rRNA (r) and ribosomal proteins (p_R)

See Supplementary Fig. 1 for a pictorial representation of the minimal microbe. See Supplementary Table 1 for a description of the model's rate constants.

Metabolism

The metabolism is made up of an extracellular substrate (s_e) which is imported by the transport enzyme (p_T) to become the intracellular species s_i. The intracellular species is converted to the universal energy substrate (e) by enzyme (p_E). Both of these enzyme mediated reactions are described by Michaelis-Menten kinetics. In addition to these processes, the intracellular substrate is diluted by cell growth ($\lambda \cdot s_i$ term in Equation 1).

$$\frac{ds_i}{dt} = \frac{v_T \cdot p_T \cdot s_e}{k_T + s_e} - \frac{v_E \cdot p_E \cdot s_i}{k_E + s_i} - \lambda \cdot s_i \tag{1}$$

Each molecule of intracellular substrate creates φ_e molecules of energy. This is a measure of the nutrient efficiency, which we maintain as high throughout (see [2] for a detailed discussion of energy implementation in their original model). Translation of each protein encoding mRNA by translation complexes consumes energy ($\sum(\dots)$ term). This energy consumption is proportional to both protein length (n_X) and number of translation complexes of that gene (c_X in the T_L function). The energy species is also diluted due to cell growth (λ).

$$\frac{de}{dt} = \varphi_e \cdot \frac{v_E \cdot p_E \cdot s_i}{k_E + s_i} - \sum_{X \in \{T,E,H,R\}} \Big(n_X \cdot \; T_L(c_X, e) \Big) - \lambda \cdot e \tag{2}$$

Host protein production

The minimal mRNA-transcriptome consists of transporter genes (denoted by T), enzymes (E), additional host proteins (H) and ribosomal protein (R). Transcription of mRNAs is modelled as a spontaneous birth process scaled by the cell's current energy status - i.e. we do not account for RNA polymerase limitation or binding kinetics but we account for the energy dependence of these processes by modifying the rate by e levels. Messenger RNAs are born at the maximal rate ω_X which is scaled by (i) any regulatory interactions $\mathcal{R}$ and (ii) energy status ($e/(o + e)$), where o is an energy threshold. The regulatory interactions are described by Supplementary Equation 3 for the respective species.

$$\mathcal{R} = 1, \quad X \in \{T, E, R\} \qquad \mathcal{R} = \frac{1}{(1 + p_H/k_H)^{h_H}}, \quad X = H \tag{3}$$

Upon birth, host mRNAs can reversibly bind/unbind free host ribosomes (R) to form translation complexes (c_X). Upon the termination of translation, mRNAs are released ($T_L(c_X, e)$ term, see Supplementary Equation

6). mRNAs are also subject to decay (δ_{m_X}) and dilution due to growth (λ).

$$\frac{dm_X}{dt} = \omega_X \cdot \mathcal{R} \cdot \Big(\frac{e}{o_X + e}\Big) + T_L(c_X, e) - b_X \cdot R \cdot m_X + u_X \cdot c_X + (\delta_{m_X} + \lambda) \cdot m_X \tag{4}$$

The dynamics of the translation complex (c_X) follow from the description of the ribosome-mRNA interactions above, with the added degradation and dilution terms:

$$\frac{dc_X}{dt} = b_X \cdot R \cdot m_X - u_X \cdot c_X - T_L(c_X, e) - (\delta_R + \lambda)c_X \tag{5}$$

The T_L function describes the rate of translation of gene X. This expression, derived in [2], relates the translation rate of individual genes to the cell's global translation rate (γ_{max} term), the gene's length (n_X) and the number of complexes currently translating that gene's mRNA (c_X).

$$T_L(c_X, e) = \frac{1}{n_X} \cdot \Big(\frac{\gamma_{max} \cdot e}{k_\gamma + e}\Big) \cdot c_i \tag{6}$$

Host proteins are born from translation complexes and are subject to decay and dilution:

$$\frac{dp_X}{dt} = T_L(c_X, e) - (\delta_{p_X} + \lambda) \cdot p_X \tag{7}$$

Note that this expression does not include the refinement of the 'empty' ribosome, p_R, into functional host ribosomes R, and so only describes the production of the protein component (see Supplementary Equation 9 for a full description of the dynamics when $X = R$).

Note also we have not included expressions describing the binding/unbinding of proteins to promoters to exert their action but rather use Hill functions to scale the mRNA production rate. For example, when we consider the auto-inhibitory effect of host proteins (i.e. when $X = H$) the derivative dp_H/dt follows the same form as dp_X/dt, and the interaction effect is only seen in dm_H/dt, i.e. the effect of the $\mathcal{R}$ term in Supplementary Equation 4. There are no terms describing the interaction of the transcription factor and promoter as there would be in a detailed mechanistic model.

Ribosome biosynthesis

As briefly described in the main text, we refined the original ribosome synthesis reactions to include the separate production of protein and rRNA based components. For the protein components, we consider the

production of a single large protein which represents the small and large ribosomal subunits and any accessory protein complexes.

We assume that host rRNAs are born spontaneously at a maximal rate scaled by the cell's internal energy, in a manner similar to the host mRNAs. This rRNA (r) reversibly binds empty ribosomes (p_R) to form free host ribosomes (R). The rRNA is subject to degradation and dilution. The dynamics of the host rRNA are therefore:

$$\frac{dr}{dt} = \omega_r \cdot \left(\frac{e}{o_r + e}\right) - b_r \cdot p_R \cdot r + u_r \cdot R - (\delta_r + \lambda) \cdot r \tag{8}$$

As previously described the protein component of the ribosome (p_R) follows the same dynamics as for other host proteins, as outlined in subsection Host protein production when $X = R$. In addition to the production and decay dynamics described in Supplementary Equation 7, p_R undergoes processing by binding with the rRNA to produce the final functional free host ribosomes, R. We account for ribosome complex disassembly by allowing this reaction to be reversible.

$$\frac{dp_R}{dt} = T_L(c_R, e) - (\delta_{p_R} + \lambda) \cdot p_R - b_r \cdot p_R \cdot r + u_r \cdot R \tag{9}$$

Free host ribosomes are produced by the reversible binding of host rRNA and empty ribosomes. They take part in translation of each protein coding gene ($\sum(\dots)$ term). Free ribosomes bind mRNAs to form translation complexes (the m_X term) and are produced when these complexes dissociate before ($u_X \cdot c_X$ term) or upon the termination of protein synthesis (T_L function term). Concurrently translation complexes are lost through degradation and dilution.

$$\frac{dR}{dt} = b_r \cdot p_R \cdot r - u_r \cdot R - \sum_{X \in \{T,E,H,R\}} \Big(T_L(c_X, e) - b_X \cdot R \cdot m_X + u_X \cdot c_X\Big) - (\delta_R + \lambda) \cdot R \tag{10}$$

Determination of growth rate

The growth rate is calculated within the model, thus allowing effects on host physiology to be assessed by observing the change in this one value. In this way the model captures the effect of high exogenous protein production such as decreasing growth rate leading to the accumulation of intermediates. Growth rate (λ) is proportional to the product of the global translation rate (γ_{max} term) and number of translating complexes

$(\sum(c_X))$. See [2] for a full derivation in terms of protein production and dilution.

$$\lambda = \frac{1}{M} \cdot \left(\frac{\gamma_{max} \cdot e}{k_\gamma + e}\right) \cdot \sum_{X \in \{T,E,H,R\}} \Big(c_X\Big) \tag{11}$$

Introduction of circuit genes

We introduce circuit genes by introducing new species and equations describing the production of mRNA, translation complexes and proteins. We assign these genes to the set Y. These take the same form as in Supplementary Equation 4, Supplementary Equation 5 and Supplementary Equation 7 with the ribosome pool specified as either host or orthogonal as appropriate. Orthogonal ribosomes are specified by modifying R to P.

We modify Supplementary Equation 2 to take account of the additional energy demand due to the circuit protein production ($\sum(\ldots)$ term).

$$\frac{de}{dt} = \varphi_e \cdot \frac{v_E \cdot p_E \cdot s_i}{k_E + s_i} - \sum_{X \in \{T,E,H,R\}} \Big(n_X \cdot\ T_L(c_X, e)\Big) - \lambda \cdot e - \sum_{Y} \Big(n_Y \cdot\ T_L(c_Y, e)\Big) \tag{12}$$

We modify Supplementary Equation 10 to take into account the additional host ribosome utilisation by circuit genes, if any, ($\sum(\ldots)$ term). If circuit genes are translated by the orthogonal ribosome pool then Supplementary Equation 17 is modified instead.

$$\begin{aligned} \frac{dR}{dt} = b_r \cdot p_R \cdot r - u_r \cdot R\ - \sum_{X \in \{T,E,H,R\}} \Big(T_L(c_X, e) - b_X \cdot R \cdot m_X + u_X \cdot c_X\Big) - (\delta_R + \lambda) \cdot R \ldots \\ - \sum_{Y} \Big(T_L(c_Y, e) - b_Y \cdot R \cdot m_Y + u_Y \cdot c_Y\Big) \end{aligned} \tag{13}$$

We modify the growth rate Supplementary Equation 11 to include the effect of circuit gene translation complexes ($\sum(c_Y)$ term):

$$\lambda = \frac{1}{M} \cdot \left(\frac{\gamma_{max} \cdot e}{k_\gamma + e}\right) \cdot \left(\sum_{X \in \{T,E,H,R\}} \Big(c_X\Big) + \sum_{Y} \Big(c_Y\Big)\right) \tag{14}$$

Addition of orthogonal ribosomes

We assume that the orthogonal 16S rRNA (ρ) follows the same dynamics as the host rRNA being produced in an energy dependent manner and reacting with empty ribosomes. We assume that this plasmid-carried gene will respond to energy changes in a manner similar to host genes (See parametrisation in Table 1).

$$\frac{d\rho}{dt} = \omega_\rho \cdot \left(\frac{e}{o_\rho + e}\right) - b_\rho \cdot p_R \cdot \rho + u_\rho \cdot P - (\delta_\rho + \lambda) \cdot \rho \tag{15}$$

We modify Supplementary Equation 10 to take account of the o-rRNA - empty ribosome interactions which mirror the host rRNA interactions (red terms). We assume that these interactions have the same kinetics as the host (See Supplementary Table 1).

$$\frac{dp_R}{dt} = T_L(c_R, e) - (\delta_{p_R} + \lambda) \cdot p_R - b_r \cdot p_R \cdot r + u_r \cdot R - b_\rho \cdot p_R \cdot \rho + u_\rho \cdot P \tag{16}$$

In the same manner as host ribosomes, free orthogonal ribosomes are produced by reversible binding of orthogonal rRNA and empty ribosomes. Functional orthogonal ribosomes bind and translate mRNAs which are specified to them (i.e. circuit genes, denoted by Y) in the same manner as host ribosomes do host mRNAs. Free ribosomes are subject to degradation and dilution due to growth.

$$\frac{dP}{dt} = b_\rho \cdot p_R \cdot \rho - u_\rho \cdot P - \sum_Y \Big(T_L(c_Y, e) - b_Y \cdot P \cdot m_Y + u_Y \cdot c_Y\Big) - (\delta_R + \lambda) \cdot P \tag{17}$$

Introduction of the controller

To implement the feedback controller in the model we introduce the new equations and species required to describe its mRNA, translation complex and protein. We denote these components with F. As described in th subsection Introduction of circuit genes, we modify the expressions for the energy species de/dt, orthogonal ribosomes dP/dt and growth rate λ to take account of the additional energy usage and translation complexes. To implement (i.e. 'close') the feedback loop, we modify Supplementary Equation 15 to include the inhibitory action of the protein p_F. As before we use a simple Hill function to describe the inhibition (red term).

$$\frac{d\rho}{dt} = \omega_\rho \cdot \left(\frac{1}{(1 + p_F/k_F)^{h_F}}\right) \cdot \left(\frac{e}{o_\rho + e}\right) - b_\rho \cdot p_R \cdot \rho + u_\rho \cdot P - (\delta_\rho + \lambda) \cdot \rho \tag{18}$$

Parametrisation

We used the parameters derived by Weiße *et al.* , using the original protein-only ribosome production parameters for the new empty ribosome (x_R) species. We assumed that the transcription of the host ribosomal rRNA showed the same energy dependence as the protein component (i.e. $\theta_r = \theta_R$). See Supplementary Table 1 for full details.

We optimised the host rRNA gene maximum transcription rate (ω_r) using MATLAB's *genetic algorithm* using 0 and 10^5 as the lower and upper bounds respectively. We choose our upper bound by assuming that maximal gene expression for a protein encoding gene is on the order of 10^3 RNAs per min and as rRNAs are present in multiple copy number and also driven from strong promoters we allow ω_r to vary significantly above that ω for a protein coding gene.

The original model was parametrised using the growth rate and ribosomal mass fraction data produced by Scott *et al.* [1, 2]. The association between growth rate (λ) and ribosomal mass fraction (Φ) was determined by growing cells on different carbon sources and in the presence of a ribosome inhibitor. We take the same approach described in [2] by incorporating the presence of inhibited ribosomes which cannot translate and by varying nutrient efficiency (φ_e). Our optimisation aims to minimise the sum of the squared errors between the data and our simulation (Supplementary Equation 19).

$$\text{cost} = \sum_{\varphi_e,\ c_0} \Big((\lambda_{sim} - \lambda_{exp})^2 + (\Phi_{sim} - \Phi_{exp})^2 \Big) \tag{19}$$

The full host model shows good quantitative agreement with the data for cellular growth rate (Supplementary Fig. 2). The simulations of the ribosomal mass fraction replicate the general trends observed in the data with ribosomal fraction increasing with antibiotic concentration and nutrient quality. There is good quantitative agreement with the experimental data at high levels of nutrient quality and low antibiotic concentration which were used in this study.

Numerical methods

Simulations

The system of ordinary differential equations was implemented in MATLAB 2016a (The MathWorks Inc., MA, USA) and its behaviour simulated using the in-built stiff solver *ode15s*. All simulations were initiated

with RNAs and translation complexes as zero and protein species at 10 molecules and are then run to steady state by repeatedly increasing the simulation time span until the maximum absolute value of the derivative vector was less than 1 unit per time unit. Before simulations of the circuit, the host model, with orthogonal ribosomes as appropriate, was simulated to steady state. All simulations were initiated with 10 molecules of each protein species and 10^3 molecules of energy.

Circuit design process

Gene circuits were designed by fixing circuit topologies and varying parameters in biologically feasible ranges. To achieve specific behaviours, we utilised the *genetic algorithm* functions from MATLAB's Global Optimisation Toolbox (version 3.4), utilising the Parallel Computing Toolbox (version 6.8) where appropriate. We optimised parameters to minimise the value of the coupling, Supplementary Equation 21.

We wished our protein outputs to mirror the behaviour of the mRNA inputs. During the circuit design processes we scored coupling between two genes by simulating the increasing production of one gene (by increasing ω_1 from 1 to 10^4) while maintaining the production of a second (ω_2 is constant). We quantified coupling by taking logs (base 10) of the induction of the first gene (ω_1) and the protein levels (p_1 and p_2) to produce the transformed data w, x and y. Using the inbuilt *polyfit* function we fit lines through the points w v x and w v y. We assessed the effect of increased w (i.e. increasing x) on y by observing the change in the gradient of this line ($y_{gradient}$). We assess the effect of the resource competition imposed by the constitutive gene on the induced gene by observing the deviation of the simulated values x (x_{sim}) from those expected by fitting a line through the points w v x (x_{fit}). Individual simulations were scored as outlined in Supplementary Equation 20:

$$\text{score}(\boldsymbol{\omega_1}, \omega_2) = \sum \left((x_{fit} - x_{sim})^2 \right) + \sum \left((y_{gradient})^2 \right) \tag{20}$$

We assessed the efficiency of a particular method to decouple two genes by calculating the sum of scores over a range of inductions of protein p_2 (vectors of induction, $\boldsymbol{\omega_2}$) (Supplementary Equation 21).

$$\text{cost}(\boldsymbol{\omega_1}, \boldsymbol{\omega_2}) = \sum_{1}^{N} \text{score}(\boldsymbol{\omega_1}, \omega_2) \tag{21}$$

We utilise this method rather than minimising the isocost line gradient as this allows us to identify designs which successfully insulate the constitutive gene whilst not necessarily relieving the resource limitation which can create a saturating response in the induced gene.

Supplementary Note 2

Impact of orthogonal ribosome usage on host physiology

To assess the impact of orthogonal ribosome production on host physiology and to test the ability of our model to capture previously reported qualitative behaviour of o-ribosome producing cells, we simulated the production of o-rRNAs and the use of o-ribosomes for gene expression (Supplementary Fig. 3).

We initially consider the production of o-ribosomes on the host cell in the absence of circuit gene expression (Supplementary Fig. 3A). We simulated the production of o-rRNAs over a number of orders of magnitude. Our results recreate previous experimental results (e.g. [11, 12]) that o-ribosome production has little effect on growth rate, with up to 20% of the ribosome pool being coopted resulting in less than a 10% fall in growth rate. Analysis of the ribosomal biosynthesis reactions (assessing rRNAs, free ribosomes etc.) shows that the 'empty ribosome' (p_R) fraction decreases significantly. Concurrently, host 16S rRNAs and mRNAs needed for ribosome assembly increase in response to ribosome sequestration by orthogonal rRNAs. The cell is able to compensate for o-ribosome sequestration by increasing the total number of ribosomes by 20%. This allows the number of orthogonal ribosomes to rise by 40% of the original ribosome number (i.e. number of host ribosomes when $\omega_\rho = 0$) whilst the number of host ribosomes falls by only 20%.

We initially simulated the expression of a simple one gene circuit which is induced at $\omega_\rho = 100$ mRNAs per minute and utilises host ribosomes for its gene expression. At this level of expression, protein output p_G is approximately 25% of the total proteome and the growth rate is predicted to be 0.024 per hour (corresponding to a doubling time of approximately 80 minutes). This is comparable to the observations of Scott *et al.* [1]. We assess the impact of orthogonal ribosome production on this gene by increasing ω_ρ over several orders of magnitude. Our model predicts a negligible fall ($< 5\%$ at $\omega_\rho = 1000$) in expression as orthogonal ribosome production increases. We do not see an increase in orthogonal ribosome production at low ω_ρ as induction of o-rRNA increases, as expected. Given the high level of predicted protein expression, there is a concurrent decrease in transcription rate due to its energy dependence (the $(e/(o_\rho + e))$ term in Supplementary Equation 15). This energy usage by protein synthesis moves the o-rRNA curve to the right and also raises the production of host rRNA and empty ribosome mRNA. If the transcription rate is increased beyond values which are biologically feasible then we observe similar effects on o-rRNA production (Supplementary Fig. 3B, inset in Host proteome).

We simulated the use of the orthogonal ribosome pool for circuit gene expression. For a constitutively expressed gene (induction held at $\omega_G = 100$) we show that protein levels, p_G, increase as o-rRNA transcription

increases, demonstrating that the size of the orthogonal ribosome pool acts as another 'dial' for controlling transgene expression (Fig. 3C). The ribosomal species, including host ribosome (R) and empty ribosomes (p_R), are most sensitive to orthogonal ribosome expression and use. Comparison of the host proteome in response to o-ribosome production (Supplementary Fig. 3A) and use (Supplementary Fig. 3C) demonstrates that use of the orthogonal ribosome pool causes more perturbation that production alone. Analysis of the ribosome biosynthesis shows that orthogonal ribosome production rises until $\omega_\rho = 40$ rRNAs per min before falling. Additionally we observe that total ribosome number falls as ω_ρ rises. This is due to the higher protein production ($> 33\%$ of the total proteome) brought about by high o-ribosome number. Translation acts to stabilise o-ribosomes, and therefore, prevents them from dissociating and releasing empty ribosomes (p_R). This means that empty ribosomes are not free to be converted into host ribosomes. This results in a decrease in the host's translational capacity, which in turn results in less translation of ribosomal mRNAs.

In our simulations, we find that at an o-rRNA induction $\omega_\rho \approx 32$ and circuit expression of $\omega_G = 100$ mRNAs per min the protein production utilising the o-ribosome is the same as when utilising the host ribosome pool (Supplementary Fig. 3C). At these equivalent levels of gene expression there is negligible change ($< 1\%$) in host protein expression ($p_T + p_E, p_H$) and growth rate (change $< 1\%$). We find significant reallocation of translational capacity with up to 25% of the ribosome pool rendered orthogonal and only negligible changes in the total number of ribosomes.

Supplementary Note 3

Crosstalk is removed in high-competition circuits

In Fig. 1B we find that as the o-ribosome pool is increased with IPTG there is a concurrent increase in h-RFP expression. This is potenally

This is likely due to incomplete orthogonality, i.e. translation of h-RFP by o-ribosomes, leading to interference. To explore this further, we introduced bi-directional crosstalk into our model, allowing o-ribosomes to translate mRNAs with host RBSs and vice versa.

To incorporate crosstalk we allow mRNAs to bind to their non-target ribosome at rate β to produce the translation complex c'. The unbinding rate is μ, which we set at 1 throughout. The translation complexes c' follow the same dynamics described in Supplementary Equation 5.

The dynamics of host-translated mRNAs m_X and proteins p_X become (with the crosstalk modifications shown in red):

$$\begin{aligned}\frac{dm_X}{dt} = \omega_X \cdot \mathcal{R} \cdot \left(\frac{e}{o_X + e}\right) \ldots \\ + T_L(c_X, e) - b_X \cdot R \cdot m_X + u_X \cdot c_X \ \ldots \\ + T_L(c'_X, e) - \beta \cdot P \cdot m_X + \mu \cdot c'_X \ \ldots \\ + (\delta_{m_X} + \lambda) \cdot m_X\end{aligned} \tag{22}$$

$$\frac{dp_X}{dt} = T_L(c_X, e) + T_L(c'_X, e) - (\delta_{p_X} + \lambda) \cdot p_X \tag{23}$$

We also modified the energy (Supplementary Equation 12) and growth rate (Supplementary Equation 14) equations to include the energy consumption and translation rates of the new complexes, c'. We included the additional mRNA-ribosome interactions by updating the equations describing the free ribosomes dynamics (Supplementary Equation 13, Supplementary Equation 17) with the modifications shown in Supplementary Equation 22 with signs inverted.

To assess the impact of circuit demand on crosstalk we simulate the production of a single protein which utilises the host ribosome pool for its expression while varying β to control the propensity of o-ribosomes to translate host and circuit mRNAs. We vary the circuit demand by changing both the translation rate (ω_{RFP}) and RBS strength (b_{RFP}). Our simulations show that in the presence of a low demand circuit, crosstalk can have a significant effect. When $\beta = b_{RFP} = 0.1$, i.e. there is no distinction between the two ribosome pools,

the protein production increases fourfold at maximium o-rRNA transcription ($\omega_{GFP}\rho = 10^3$). As o-ribosome production is increased by increasing ω_ρ there is a net increase in ribosome number (Supplementary Fig. 3B). These results suggest that the h-RFP circuit, which contains only one gene that has low demand for ribosomes and so crosstalk results in increased h-RFP expression as o-ribosomes are produced (Supplementary Fig. 5B).

Increasing the circuit demand by increasing both ω_{RFP} and b_{RFP} to their maximum feasible values effectively removes the impact of crosstalk with protein levels falling negligibly (Supplementary Fig. 5C). Comparing our high demand simulations to the experimental results of the h-RFP, h-GFP circuit shows that our gene coupling assessments are carried out in a context of high enough competition to allow crosstalk to be considered negligible throughout (Supplementary Fig. 5D) and therefore throughout our experimental system.

Supplementary Note 4

Inefficient o-ribosome assembly may explain poor gene expression from the o-system

Our simulations predict that for a given gene induction, utilisation of the orthogonal ribosome pool results in higher protein production due to the lack of competition (Supplementary Fig. 3). However, experimentally we find significantly reduced expression (Fig. 1).

This may be due to lower production of the o-rRNA in comparison to the host rRNA operons but at moderate to high levels of o-rRNA induction and (assuming $b_G = 1$ and $b_\rho = 1$, i.e. a strong orthogonal RBS and complete assembly) simulations suggest that use of the orthogonal ribosome pool produces higher gene expression than the host. Therefore, we propose that our experimental observations may be due to inefficient translation (i.e. the orthogonal RBS is weak) or inefficient orthogonal ribosome assembly. To assess these effects we simulate the production of a single protein (induced at $\omega_G = 100$ molecules per min) using both the host ribosome pool (with maximal RBS strength $b_G = 1$) and o-ribosome pool. In the latter simulations, we vary the mRNA-ribosome association rate ($b_G < 1$) and the o-rRNA-empty ribosome association rate ($b_\rho < 1$). We leave the unbinding rates as maximal, i.e. $u_G = 1$ and $u_\rho = 1$ and set the maximum o-rRNA rate $\omega_\rho = 100$ molecules per min.

Varying each parameter alone is sufficient to cause decrease in gene expression to less than 40% of the production achieved using the host pool. If both b_G and b_ρ are significantly weaker than those of the host ($b_G < 0.1$ and $b_\rho < 0.1$, Fig. 7) then the ratio of the protein production using the orthogonal to the host pool falls to ranges we see experimentally.

Supplementary Note 5

Competition for 'empty ribosomes' explains increased coupling observed in o-RFP, h-GFP circuits

Given the increase in coupling observed in the o-RFP, h-GFP arrangement, we assessed the change in protein components. As the induction of o-RFP is increased (simulated by increases ω_{RFP}), it forms translation complexes with the o-ribosomes. This stabilises their formation, preventing release of empty ribosomes. We observe marked declines in empty ribosomes, i.e. the protein core which is not specified until bound by an rRNA. This results in a concurrent fall in host ribosome number (both free, R and translation complexes $h - c_X$). This results in significant perturbation of host protein levels when the production of o-ribosomes is high (Supplementary Fig. 16, $\omega_\rho = 500$). This in turn results in a concurrent fall in expression of the host ribosome-utilising GFP by over 80% at medium levels of RFP induction ($\omega_{RFP} = 100$, $\omega_\rho = 500$ molecules per min.)

Supplementary Note 6

Multiple orthogonal ribosome pools decouple co-expressed genes

In the main text we demonstrate both numerically and experimentally that utilising both the host and orthogonal ribosomes pools alters gene coupling profiles. We extend this analysis by considering the effect of using two orthogonal ribosome pools. Biologically these can be created by expressing multiple different synthetic 16S rRNAs [11]. We implement a second orthogonal ribosome pool by replicating the changes outlined in Supplementary Note 1. At high levels of o-rRNA expression, competition for empty ribosomes (p_R) results in high levels of gene coupling.

We optimise the production of the two orthogonal 16S rRNAs to minimise gene coupling ($\omega_{\rho_1} = \omega_{\rho_2} = 10$ molecules per min) which yields near complete decoupling. However this assumes that there is no cross talk between the two orthogonal ribosome pools - i.e. that each pool translates its, and only its, target mRNAs. In reality, to date, orthogonal rRNA sequences are often not sufficiently distinct to achieve this and each orthogonal pool will translate mRNAs targeted for the other pool due to cross talk - i.e. erroneous interactions between one 16S rRNA and its non-target mRNA. To test the impact of cross talk we introduce interactions between each mRNA and its non-target o-ribosome, as in Section Supplementary Note 3. Messenger RNAs bind to their non-target o-ribosome at rate β to produce the translation complex c_1'. The unbinding rate is μ. The dynamics of an mRNA m_1 which is designed to be translated by P_1 and erroneous translated by P_2 (the effect of crosstalk is highlighted in red) are:

$$\frac{dm_1}{dt} = \omega_1 \cdot \left(\frac{e}{o_1 + e}\right) + T_L(c_1, e) - b_1 \cdot P_1 \cdot m_1 + u_1 \cdot c_1 + T_L(c_1', e) - \beta_1 \cdot P_2 \cdot m_1 + \mu_1 \cdot c_1' - (\delta_{m_1} + \lambda) \cdot m_1 \quad (24)$$

We vary the level of crosstalk between none ($\beta = 0$) and high ($\beta = 0.1$). Coupling increases seven fold as crosstalk increases from 0 to 0.1 (the second represents 10% of the interaction between o-ribosome and its target mRNA). As cross talk increases, gene coupling (as measured by the slope of the isocost line) also increases (Supplementary Fig. 19B). Whilst our simulations suggest that some level of crosstalk can be tolerated, this must be relatively small ($\beta \leq 0.01$), and may not be achievable with currently published o-ribosomes.

Supplementary Note 7

The use of tethered o-ribosomes may increase yields and increase decoupling in some contexts

So far our analysis has focused on the assessing the feasibility using synthetic 16S rRNAs to produce a synthetic orthogonal ribosomes. These ribosomes function in the same manner as natural host ribosomes with altered specificity; for example, these o-ribosomes dissociate into their respective large and small subunits when not translating. By expressing synthetic 23S rRNAs or additional synthetic RNAs then 'tethered' ribosomes can be created [13, 14]. In these semi-synthetic ribosomes the two subunits are permanently linked and so are not able to dissociate.

Here we assess the effect of using tethered ribosomes for synthetic gene expression and its impact on gene coupling by simulating simple two-gene circuits. Ribosome tethering can be modelled by assuming that the o-rRNA and protein components could not dissociate upon creation of the functional o-ribosome, i.e. we set u_ρ to 0. We set $b_\rho = 0.45$ to take into account the experimental observation that tethered ribosomes shown reduced expression in comparison to host ribosomes [13].

The expression of tethered ribosomes results significant decrease in expression of circuits using the host ribosome pool due to the lack of recycling of the ribosomal proteins p_R (Supplementary Fig. 20A). Utilising the tethered o-ribosome pool for gene expression significantly increases gene expression from the circuit (Supplementary Fig. 20B). Simulating a circuit when the two circuits genes are distributed across both host and tethered o-ribosome pools replicates results observed with orthogonal ribosomes. When the constitutively expressed gene is translated by the tethered o-ribosome pool and the induced gene by the host pool we observe complete decoupling and increased gene expression at all levels of o-ribosome pool (Supplementary Fig. 20C). The opposite arrangement significantly increases coupling (Supplementary Fig. 20D).

Supplementary Note 8

Gene expression plateauing is mediated by saturation of the ribosome pool

As RFP induction increases the output levels begin to plateau (both experimentally Fig. 2c,3c and theoretically Fig. 5). To assess the potential reasons for this saturation effect we simulate the different circuit conformations and assess the mRNA (input) – protein(output) relationship. The internal intermediate, e, does not fall to below 85% across all inductions (Supplementary Fig. 24). This metabolite represents the outputs of metabolism; such as 'energy' (approximating the function ATP/GTP) but also the precursers needed for protein synthesis (such as amino acids). As this metabolite is well maintained across inductions we propose that this is not the cause of the saturation effect. The transcription rates in our model are not limiting with GFP mRNA outputs being maintained across RFP inductions and RFP mRNAs increasing linearly with induction. Whilst our model does not explicitly include RNA polymerase sharing across the genes the propensity for genes to be expressed under different energy conditions, and therefore different make ups of the cellular economy, is included via the transcriptional thresholds (θ terms). See [2] for original discussion. Therefore we propose that the saturation effect observed in our experimental system remains due to reduced free ribosome number rather than other factors.

Supplementary Note 9

Modelling of a biochemical pathway

We consider a simple five-part linear biochemical pathway which converts the intracellular 'energy' metabolite, e, to an output substrate, s_5, via four intermediates (Supplementary Equation 25).

$$e \xrightarrow{v_1,\ k_1} s_1 \xrightarrow{v_2,\ k_2} s_2 \xrightarrow{v_3,\ k_3} s_3 \xrightarrow{v_4,\ k_4} s_4 \xrightarrow{v_5,\ k_5} s_5 \tag{25}$$

We model the conversion of each metabolite, s_i, into the next as an enzyme catalysed reaction by protein p_{i+1} with the reaction dynamics described by Michaelis-Menten kinetics.

The dynamics of the first metabolite are:

$$\frac{ds_1}{dt} = \frac{v_1 \cdot e \cdot p_1}{k_1 + e} - \frac{v_2 \cdot s_1 \cdot p_2}{k_2 + s_1} - \lambda \cdot s_1 \tag{26}$$

The dynamics of the intermediate metabolites are (where $i = \{2...4\}$):

$$\frac{ds_i}{dt} = \frac{v_i \cdot s_{i-1} \cdot p_i}{k_i + s_{i-1}} - \frac{v_{i+1} \cdot s_i \cdot p_{i+1}}{k_{i+1} + s_i} - \lambda \cdot s_i \tag{27}$$

The dynamics of the final metabolite, the output, are defined by:

$$\frac{ds_5}{dt} = \frac{v_5 \cdot s_4 \cdot p_5}{k_5 + s_e} - \lambda \cdot s_5 \tag{28}$$

We also modify Supplementary Equation 12 to account for the conversion of e into s_1. This represents the drain on the host metabolic flux of the new pathway. φ_s represents the number of molecules of e required to make one molecule of s_1.

$$\frac{de}{dt} = \varphi_e \cdot \frac{v_E \cdot p_E \cdot s_i}{k_E + s_i} - \sum_{X \in \{T,E,H,R,Y\}} \Big(n_X \cdot\ T_L(c_X, e) \Big) - \lambda \cdot e \, {\color{red} - \varphi_s \cdot \frac{v_1 \cdot e \cdot p_1}{k_1 + p_1}} \tag{29}$$

We model the production of the enzymes as we modelled the reporter genes before (described in Supplementary Note 1). Whilst, experimentally *vioBCDE* is expressed as an operon from one promoter, for simplicity we model these genes as four separate mRNAs (i.e. one mRNA for *vioB* etc) to maintain the number of

RBSs and hence competition. We ensure the parameters representing the mRNA birth-death processes are equal (i.e. ω_i, o_i and δ_{m_i} for $i = \{2...5\}$) to model the fact these genes are co-regulated.

We assumed that pathway enzymes have the same kinetics as the host's lumped enzyme function ($v_i = 5800$ molecules per min and $k_i = 1000$ molecules). We set φ_s to be 0.01 to model the impact of the additional flux through the pathway on the central metabolism. We also increase the nutrient efficiency to φ to 1 to model the additional media supplementation. We take the enzyme sizes, in amino acids, for the components of the violacein producing pathway from UniProt: $n_1 = 418, n_2 = 998, n_3 = 429, n_4 = 373, n_5 = 196$. Other parameters, where set as found in Supplementary Table 2. Note that for simplicity of language and to allow the same nomenclature with the experimental implementation of the violacein pathway the enzymes are referred to by the a letter in the main text, such that $A = 1$, $B = 2$ etc.

References

[1] M. Scott, C. W. Gunderson, E. M. Mateescu, Z. Zhang, and T. Hwa, "Interdependence of cell growth and gene expression: origins and consequences.," *Science*, vol. 330, no. 6007, pp. 1099–1102, 2010.

[2] A. Y. Weiße, D. A. Oyarzún, V. Danos, and P. S. Swain, "Mechanistic links between cellular trade-offs, gene expression, and growth," *Proceedings of the National Academy of Sciences*, vol. 112, no. 9, pp. E1038–E1047, 2015.

[3] A. Sachdeva, K. Wang, T. Elliott, and J. W. Chin, "Concerted, rapid, quantitative, and site-specific dual labeling of proteins," *Journal of the American Chemical Society*, vol. 136, no. 22, pp. 7785–7788, 2014.

[4] F. R. Blattner, G. Plunkett, C. A. Bloch, N. T. Perna, V. Burland, M. Riley, J. Collado-Vides, J. D. Glasner, C. K. Rode, G. F. Mayhew, J. Gregor, N. W. Davis, H. A. Kirkpatrick, M. A. Goeden, D. J. Rose, B. Mau, and Y. Shao, "The Complete Genome Sequence of Escherichia coli K-12," *Science*, vol. 277, no. 5331, 1997.

[5] D. Hanahan and M. Meselson, "Plasmid screening at high colony density," *Gene*, vol. 10, no. 1, pp. 63–67, 1980.

[6] E. Martínez-García and V. de Lorenzo, "Engineering multiple genomic deletions in Gram-negative bacteria: Analysis of the multi-resistant antibiotic profile of Pseudomonas putida KT2440," *Environmental Microbiology*, vol. 13, no. 10, pp. 2702–2716, 2011.

[7] A. Gyorgy, J. I. Jiménez, J. Yazbek, H.-H. Huang, H. Chung, R. Weiss, and D. Del Vecchio, "Isocost Lines Describe the Cellular Economy of Genetic Circuits," *Biophysical Journal*, vol. 109, no. 3, pp. 639–646, 2015.

[8] R. Silva-Rocha, E. Martínez-García, B. Calles, M. Chavarría, A. Arce-Rodríguez, A. de Las Heras, A. D. Páez-Espino, G. Durante-Rodríguez, J. Kim, P. I. Nikel, R. Platero, and V. de Lorenzo, "The Standard European Vector Architecture (SEVA): a coherent platform for the analysis and deployment of complex prokaryotic phenotypes.," *Nucleic Acids Research*, vol. 41, no. Database issue, pp. D666–75, 2013.

[9] K. Datsenko and B. Wanner, "One-step inactivation of chromosomal genes in Escherichia coli K-12 using PCR products," *Proceedings of the National Academy of Sciences USA*, vol. 97, no. 12, pp. 6640–6645, 2000.

[10] K. Wang, A. Sachdeva, D. J. Cox, N. W. Wilf, K. Lang, S. Wallace, R. A. Mehl, and J. W. Chin, "Optimized orthogonal translation of unnatural amino acids enables spontaneous protein double-labelling and FRET," *Nature Chemistry*, vol. 6, no. 5, pp. 393–403, 2014.

[11] O. Rackham and J. W. Chin, "A network of orthogonal ribosome·mRNA pairs," *Nature Chemical Biology*, vol. 1, no. 3, pp. 159–166, 2005.

[12] L. M. Chubiz and C. V. Rao, "Computational design of orthogonal ribosomes," *Nucleic Acids Research*, vol. 36, no. 12, pp. 4038–4046, 2008.

[13] C. Orelle, E. D. Carlson, T. Szal, T. Florin, M. C. Jewett, and A. S. Mankin, "Protein synthesis by ribosomes with tethered subunits," *Nature*, vol. 524, pp. 119–124, 2015.

[14] S. D. Fried, W. H. Schmied, C. Uttamapinant, and J. W. Chin, "Ribosome subunit stapling for orthogonal translation in E. coli," *Angewandte Chemie - International Edition*, vol. 54, no. 43, pp. 12791–12794, 2015.
